# Seeding activity of skin misfolded tau as a biomarker for tauopathies

**DOI:** 10.1186/s13024-024-00781-1

**Published:** 2024-11-29

**Authors:** Zerui Wang, Ling Wu, Maria Gerasimenko, Tricia Gilliland, Zahid Syed Ali Shah, Evalynn Lomax, Yirong Yang, Steven A. Gunzler, Vincenzo Donadio, Rocco Liguori, Bin Xu, Wen-Quan Zou

**Affiliations:** 1 Department of Pathology, Case Western Reserve University School of Medicine, Cleveland, OH USA; 2 Biomanufacturing Research Institute and Technology Enterprise, North Carolina Central University, Durham, NC USA; 3 Institute of Neurology, Department of Neurology, Jiangxi Academy of Clinical Medical Sciences, Rare Disease Center, Key Laboratory of Rare Neurological Diseases of Jiangxi Province Health Commission, The First Affiliated Hospital, Jiangxi Medical College, Nanchang University, Nanchang, Jiangxi Province China; 4 Neurological Institute, University Hospitals Cleveland Medical Center, Cleveland, OH USA; 5 Department of Neurology, Case Western Reserve University School of Medicine, Cleveland, OH USA; 6 IRCCS Institute of Neurological Sciences of Bologna, UOC Clinica Neurologica, Bologna, Italy

**Keywords:** Tauopathies, Alzheimer’s disease, Seeding activity, Tau, Real-time quaking-induced conversion (RT-QuIC), Skin

## Abstract

**Background:**

Tauopathies are a group of age-related neurodegenerative diseases characterized by the accumulation of pathologically hyperphosphorylated tau protein in the brain, leading to prion-like aggregation and propagation. They include Alzheimer’s disease (AD), progressive supranuclear palsy (PSP), corticobasal degeneration (CBD), and Pick’s disease (PiD). Currently, reliable diagnostic biomarkers that directly reflect the capability of propagation and spreading of misfolded tau aggregates in peripheral tissues and body fluids are lacking.

**Methods:**

We utilized the seed-amplification assay (SAA) employing ultrasensitive real-time quaking-induced conversion (RT-QuIC) to assess the prion-like seeding activity of pathological tau in the skin of cadavers with neuropathologically confirmed tauopathies, including AD, PSP, CBD, and PiD, compared to normal controls.

**Results:**

We found that the skin tau-SAA demonstrated a significantly higher sensitivity (75–80%) and specificity (95–100%) for detecting tauopathy, depending on the tau substrates used. Moreover, the increased tau-seeding activity was also observed in biopsy skin samples from living AD and PSP patients examined. Analysis of the end products of skin-tau SAA confirmed that the increased seeding activity was accompanied by the formation of tau aggregates with different physicochemical properties related to two different tau substrates used.

**Conclusions:**

Overall, our study provides proof-of-concept that the skin tau-SAA can differentiate tauopathies from normal controls, suggesting that the seeding activity of misfolded tau in the skin could serve as a diagnostic biomarker for tauopathies.

**Supplementary Information:**

The online version contains supplementary material available at 10.1186/s13024-024-00781-1.

## Introduction

The deposition of disease-associated tau aggregates in the brain is a characteristic feature of tauopathies, including Alzheimer’s disease (AD), progressive supranuclear palsy (PSP), corticobasal degeneration (CBD), and Pick’s disease (PiD) [1]. These conditions share a common pathogenesis involving the seeding and propagation of misfolded and hyperphosphorylated isoforms of the tau protein in the brain, reminiscent of the infectious prion protein (PrP^Sc^ or prion) in prion diseases (PrD) [2]. The human brain expresses six tau isoforms, resulting from the combination of three or four microtubule-binding repeats (3R or 4R tau) and 0–2 N-terminal inserts (0 N, 1 N, or 2 N tau) [3]. Different tauopathies exhibit variations in the composition of these tau isoforms, with AD displaying both 3R and 4R isoforms, PiD primarily containing 3R isoforms, as well as PSP and CBD characterized by the main accumulation of 4R tau assemblies. These differences in tau isoform composition are proposed to be associated with the presence of distinct strains of neurotoxic tau aggregate conformers [4]. Currently, the definitive diagnosis of tauopathies relies on the availability of brain tissues obtained via biopsy or at autopsy for the detection of tau pathology and phosphorylated tau. However, these methods are highly invasive or often too late in the disease process. Recent advancements in brain molecular imaging and highly sensitive immunoassays of phosphorylated and total tau (p-tau and t-tau) in plasma and cerebrospinal fluid (CSF) have enabled early and reliable diagnosis of AD in living patients [5], but they have limitations such as invasiveness or high cost. Newly developed single molecular immunoassay (Simoa) is highly sensitive and specific in detection of the blood biomarkers, but Simoa is unaffordable to the majority of patients, especially those in the developing countries.

The seed-amplification assay (SAA) technology, utilizing real-time quaking-induced conversion (RT-QuIC) or protein misfolding cyclic amplification (PMCA), offers an ultrasensitive approach to identify disease-specific biomarkers in easily accessible specimens for early diagnosis and disease progression assessment [6–8]. This technology has been used to detect prions and other prion-like misfolded proteins such as α-synuclein in body fluids and peripheral tissues of PrD and PD by assessing the seeding activity (SA) of misfolded proteins [9–15]. RT-QuIC has also been reported to detect tau-SA in the brain tissues of deceased individuals with tauopathies [16–20]. However, there have not been any reports on the application of tau-SAA to the body fluids and peripheral tissues of patients with AD to date although there was a report about tau RT-QuIC detection of CSF from PSP and CBD [19].

In the current study, we investigated tau-SA in autopsy scalp skin samples from individuals with neuropathologically confirmed AD, PSP, CBD, PiD, and normal controls (NC) using RT-QuIC with truncated human tau fragments as substrates. We found that misfolded tau from AD and other tauopathies like PSP and CBD, but not from NC and PiD, selectively seeded the 4RCF substrate (equivalent to the human 4R tau fragment, K18CFh) [16, 20] with a sensitivity of 80.5% and a specificity of 95.4%. Similarly, the 3RCF substrate (equivalent to the human 3R tau fragment, K19CFh) [18, 20] showed a sensitivity of 77% and a specificity of 92%. Biopsied skin samples from individuals with AD, PSP, and controls also exhibited high diagnostic efficacy. Furthermore, the study demonstrated that skin-derived misfolded tau aggregates from AD, containing a mix of 4R and 3R tau, and from PSP and CBD, with a dominant 4R tau, could be successfully amplified using either 4RCF or 3RCF substrates. In contrast, skin misfolded tau aggregates from PiD, mainly composed of 3R tau, showed amplification only with the 3RCF substrate, not with 4RCF. This study represents the first application of SAA methodology to both post-mortem and biopsied cutaneous specimens and highlights the potential of skin tau-SAA as a novel biomarker for the diagnosis of tauopathies.

## Results

### Skin tau-SAA can specifically differentiate AD and non-AD tauopathies from normal controls and PiD cases using 4RCF as the substrate

In contrast to PrP in PrD and α-synuclein in PD, the tau molecule in the human brain exhibits 6 isoforms, of which there are 3 isoforms with 4 microtubule-binding repeats (4R tau) and 3 with three repeats (3R tau), resulting from alternative mRNA splicing. In order to find an appropriate tau substrate for SAA of skin tau (sTau-SAA) with RT-QuIC, based on our recent finding with autopsy brain tissues from cadavers with AD and other non-AD tauopathies [20], we first examined 4 types of tau substrates including 2 full-length tau isoforms (2N3R/2N4R) and 2 truncated tau fragments consisting of 4RCF (cysteine-free, equivalent to K18CFh)/3RCF (equivalent to K19CFh) using autopsy skin samples from AD cadavers diagnosed neuropathologically. 4RCF represents the aggregation-prone core sequences of the 4R tau isoforms while 3RCF fragment is the aggregation-prone core sequences of the 3R tau isoforms [20, 21]. The only difference between 4RCF and 3RCF is that 3RCF lacks the R2 region. Compared to the full-length tau, the truncated tau substrates-based RT-QuIC revealed higher endpoint fluorescence readings (exceeding 100,000 RFU) and shorter lag phases (less than 25 h) (Fig. S1). As a result, we used the two truncated tau fragments as the substrates for the following examinations in our study.

**Fig. 1:**
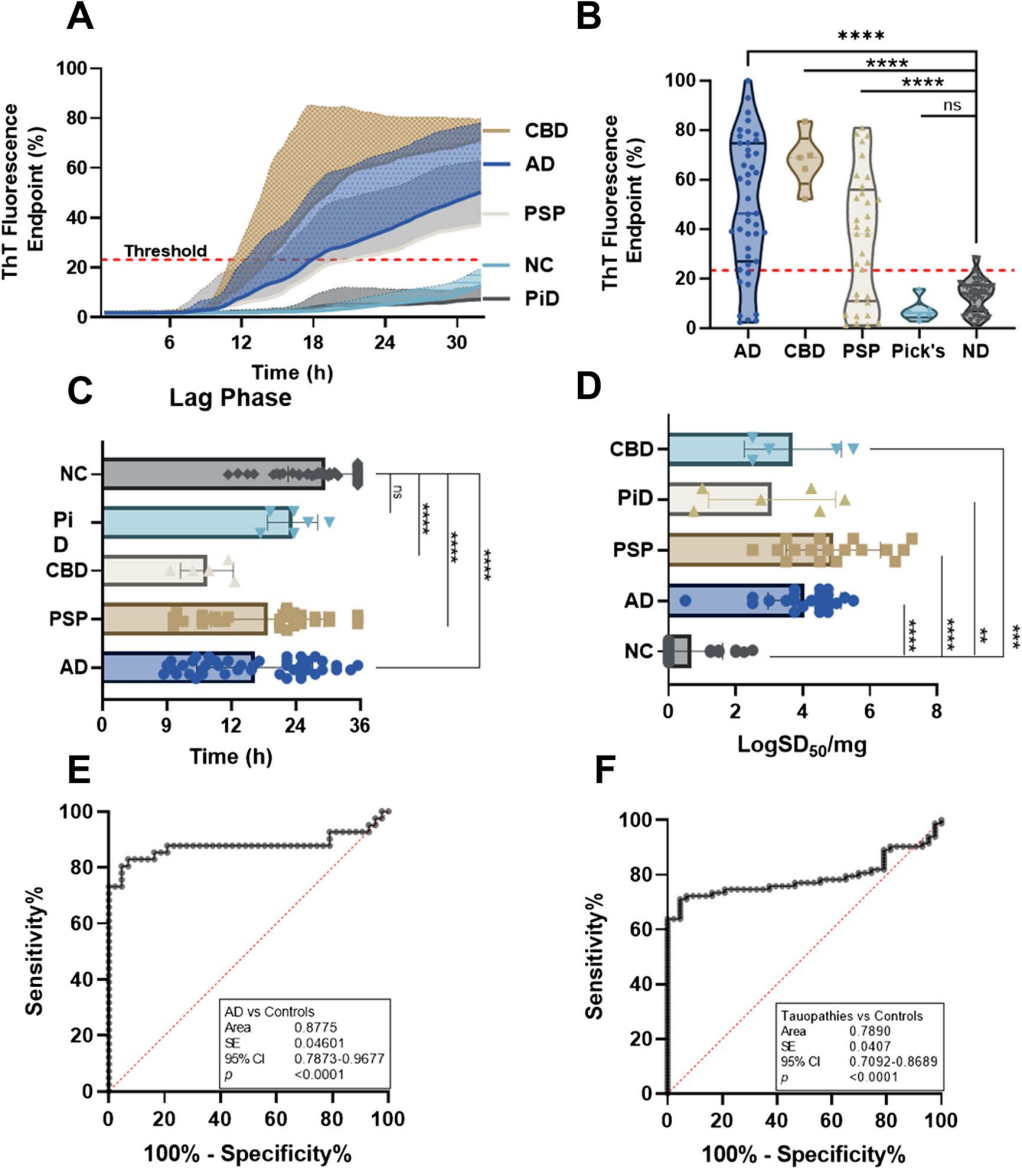
Tau-seeding activity of skin samples from patients with tauopathies using 4RCF-based RT-QuIC. **A** Kinetic curves displaying the mean and standard deviation (SD) of tau-SA over time of skin samples from CBD (*n* = 5), AD (*n* = 46), PSP (*n* = 33), PiD (*n* = 6) and NC (*n* = 43). **B** Scatter plot illustrating the distribution of tau-SA across different tauopathies detected in panel **A**. **C** Lag phase, defined as the initial period before a significant increase in the ThT fluorescence intensity. **D** The end-point dilution analysis of quantitative tau-SA of skin samples from tauopathies. The half of maximal SA (SD50) was determined by Spearman-Kärber analyses and is shown as log SD50/mg skin tissue. **E** Receiver operating characteristic (ROC) curve analysis comparing tau-SA of skin samples between AD and control subjects, with an area under the curve (AUC) of 0.82. **F** ROC curve analysis comparing tau-SA of skin samples between total tauopathy and control subjects, with an AUC of 0.79. ns: *p* > 0.05; **: *p* < 0.01; ***: *p* < 0.001; ****: *p* < 0.0001

Using RT-QuIC with 4RCF truncated tau as the substrate, we analyzed autopsy skin samples from AD (*n* = 46), CBD (*n* = 5), PSP (*n* = 33), PiD (*n* = 6), and NC (*n* = 43). 4RCF-based RT-QuIC of skin sTau-SA exhibited that CBD cases had the highest ThT fluorescence intensity, followed by AD, PSP, and PiD (Fig. 1A, B). We also compared lag phases of sTau-SA in each type of tauopathy, the time period between the beginning of the RT-QuIC measurement and the time point at which the curves reflecting the ThT fluorescence started to hit the half of the maximum of ThT fluorescence. Consistent with the endpoint ThT values, CBD displayed the shortest lag phase, while PiD was the longest (Fig. 1C). Our 4RCF-based RT-QuIC assay of the skin samples from AD and non-AD tauopathies yielded a sensitivity of 80.49% and a specificity of 95.35%. To quantitate the sTau-SA of each type of tauopathy, we conducted endpoint titration of RT-QuIC assay of each skin sample from different tauopathies. The x-axis represents SD50 that referred to the 50% seeding activity. PSP samples exhibited the highest sTau-SA, followed by AD samples. In contrast, PiD skin samples displayed markedly lower tau-SA, as shown in Fig. 1D. This observed variation is likely attributable to the incompatibility between seed and substrate, particularly noting that PiD-derived misfolded tau is mainly comprised of 3R tau. AUC analysis revealed an area value of 0.88 based on the comparison between AD and normal controls and 0.79 based on the comparison between tauopathies and normal controls (Fig. 1E, F).

### The sTau-SAA of AD and all other tauopathies can be specifically differentiated from that of normal controls using 3RCF as the substrate

**Fig. 2:**
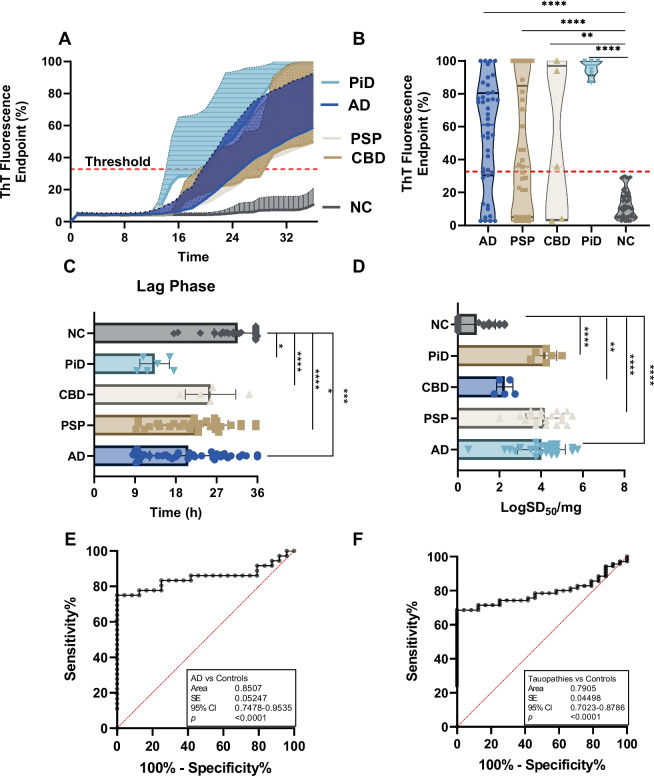
Tau-seeding activity of skin samples from patients with tauopathies using 3RCF-based RT-QuIC assay. **A** Kinetic curves displaying the mean and SD of tau-SA over time of skin samples from CBD (*n* = 5), AD (*n* = 46), PSP (*n* = 33), PiD (*n* = 6) and NC (*n* = 43). **B** Scatter plot illustrating the distribution of tau-SA across different tauopathies. **C** Comparison of lag phases of skin tau-SAA from different tauopathies and NC, same as above, as the initial delay before the ThT fluorescence intensity begins to rise. **D** The end-point dilution analysis of quantitative tau-SA of skin samples from various tauopathies. The half of maximal SA (SD50) determined by Spearman-Kärber analyses is shown as log SD50/mg skin tissue. **E** ROC curve analysis comparing skin tau-SA between AD and control subjects, with an AUC of 0.77. **F** ROC curve analysis comparing skin tau-SA between total tauopathy and control subjects, with an AUC of 0.72. *: *p* < 0.05; **: *p* < 0.01; ***: *p* < 0.001; ****: *p* < 0.0001

We next used 3RCF (K19)-based RT-QuIC assays to examine the same samples detected in 4RCF studies. The sTau-SA was significantly higher in tauopathies than in non-tauopathies, of which PiD was the highest, followed by AD, PSP, and CBD (Fig. 2A, B). We also compared their lag phases, which were inversely proportional to those of 4R tau: PiD exhibited the shortest lag phase, while CBD showed the longest lag phase (Fig. 2C). PiD and PSP demonstrated the highest seeding dose while CBD was lowest (Fig. 2D). In addition, the 3RCF-based skin tau RT-QuIC assay generated a sensitivity of 75% and a specificity of 100%, which was similar to that of 4RCF-based RT-QuIC in general. The AUC values (0.85 vs. 0.79) of 3RCF-based RT-QuIC assay of skin tau were similar to those detected with 4RCF-based RT-QuIC shown above (Fig. 2E, F). Notably, in contrast to the 4RCF-based sTau-SA, 3RCF-based sTau-SA from PiD was the highest, in addition to differentiating other tauopathies from the normal controls.

Then we compared the tau-SA of brain and skin samples from AD using the two truncated tau fragments as substrates. Tau-SA was significantly lower in skin than in brain tissues either with 4RCF [skin (*n* = 20), 182,400.8 ± 60,353.3 (mean ± SD) vs. brain (*n* = 12), 252,170.1 ± 11,147.6; *p* = 0.0034 < 0.005] or with 3RCF as the substrate [skin (*n* = 20), 177,442.3 ± 62,953.4 vs. brain (*n* = 12), 215,374.9 ± 48,246.7; *p* = 0.0061 < 0.01] (Fig. S2A, B).

The correlation of tau-SA in the skin and brain of 8 cadavers was analyzed, from whom we obtained both brain and skin tissues of the same individuals for RT-QuIC assays. There was a trend of a positive correlation of tau-SA between skin and brain tissues with both 4RCF and 3RCF substrates although they were not statistically significant (*p* > 0.05) (Fig. S2C-F).

To determine whether there is a difference in tau between brain and skin tissues, we examined the phosphorylated tau of brain and skin tissues from AD cadavers by western blotting with pS396 and pT231 antibodies against phosphorylated tau. There were significant differences in the levels and gel profiles of phosphorylated tau between the two tissues (Fig. S3A, B). We also examined the ratio of the skin 3R/4R tau isoforms by western blotting with antibodies specifically directed against 3R (RD3) and 4R (RD4) antibodies (Fig. S3C, D). By densitometry of protein bands on western blots, the ratio of 3R/4R tau in the skin was 1.3:1, which is consistent with that reported in the brain tissues of AD [22]. We performed a quantitative analysis of AD brain and skin samples from tauopathies based on the Western blot, as presented in Fig. S3E (pT231) and Fig. S3F (pS396). Our findings show that the pS396 level in the brain is approximately 160 times higher than in the skin of tauopathy patients, while the pT231 level in the brain is around 60 times higher than in the skin.

We also conducted the western blotting of skin samples from AD and other tauopathies with the anti-tau antibody HT7 (Fig. S4). Notably, HT7 detects tau bands with high molecular weights including 100 kDa and higher close to 250 kDa, greater than those of typical monomeric tau proteins. They could be the dimers or oligomers based on their molecular weights. Meanwhile, HT7 also detects small tau fragments migrating between 12 and 35 kDa, whose molecular weights are smaller than those of any individual monomers of 6 isoforms (Fig. S4). Based on their smaller molecular weights, they are most likely the truncated forms of tau isoforms.

Given that the tau-SA was not significantly different among some of tauopathies, the specificity of the amplification reaction may not be certain. To determine whether the tau-SA is indeed specifically from misfolded tau isoforms, we used tau immunodepletion (ID) by immunoprecipitation of tau with an anti-tau antibody from skin samples prior to RT-QuIC assay. We revealed that tau-SA was significantly decreased in the samples subjected to ID compared to the samples without ID [41,285 ± 20,657 (AD_ID skin, mean ± SD) vs. 149,573 ± 61,571 (AD skin), *p* < 0.0001] (Fig. S5), confirming the specificity of tau-SA by RT-QuIC.

### The sTau-SA is significantly elevated over an increase in the Braak staging

**Fig. 3:**
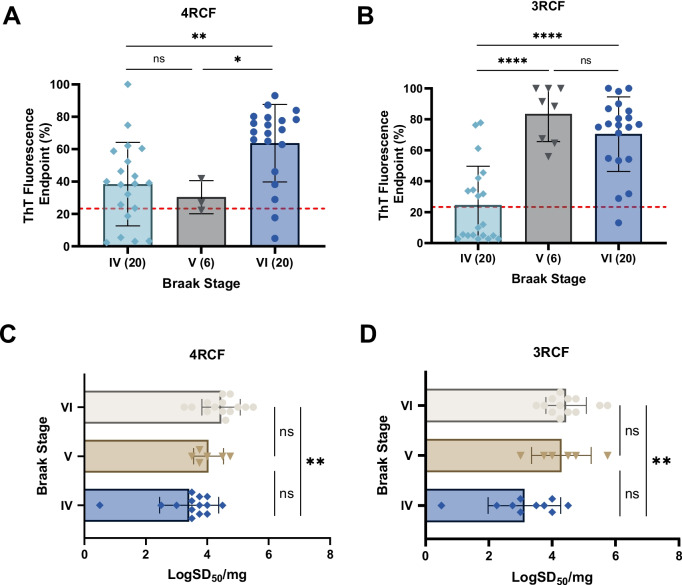
Correlation of tau-SA of skin samples from AD with their different Braak stages. Scatter plot of ThT fluorescence readings at end-points detected by RT-QuIC assays with the substrate 4RCF (**A**) or 3RCF (**B)** as a function of different Braak stages [IV (*n* = 20), V (*n* = 6) and VI (*n* = 20)]. Scatter plot of Braak stages with LogSD_50_/mg of skin tau-SA detected by RT-QuIC with the substrate 4RCF (**C**) or 3RCF (**D**), ns: *p* > 0.05; ** *p* < 0.01; ****p < 0.0001

Based on Aβ/tau-pathology and Aβ/tau-positron emission tomography (PET) scan, the accumulation of Aβ/tau leading to clinical AD is a continuum process. To determine whether the skin tau-SA can reflect the severity or Braak staging in the AD brain, next we associated skin tau-SA with the Braak staging in autopsy brain tissues examined. Notably, when sTau-SA with both 4RCF and 3RCF served as a function of Braak staging, there was a clear association observed: the sTau-SA was significantly elevated upon an increase in the Braak staging (Fig. 3). Specifically, the overall ThT intensity for 4RCF-based skin tau-SA was increased following advancing of the Braak stages, although no significant difference was noted between stages IV and V (Fig. 3A). A similar trend was observed for 3RCF, where the overall ThT intensity increased with the progression of Braak stage (Fig. 3B), yet no significant difference was found between stages V and VI.

We also explored whether variables such as age, gender, and post-mortem interval (PMI) could influence Tau-SA. We observed no significant differences between males and females in both 4RCF (Fig. S6A, B) and 3RCF (Fig. S6C, D) assays. In the correlation analysis between ThT fluorescence intensity and age/PMI of the autopsied skin tissues, no correlation was found for 4RCF with either age (Fig. S6E) (*r* = 0.07247, *p* = 0.5047 > 0.05) or PMI (Fig. S6F) (*r* = -0.02187, *p* = 0.8406 > 0.05). For 3RCF, a slight positive correlation was observed between age and Tau-SA end-point ThT fluorescence, though this was not statistically significant (Fig. S6G) (*r* = 0.1042, *p* = 0.3366 > 0.05); similarly, a slight negative correlation was noted between PMI and ThT fluorescence (*r* = -0.1225, *p* = 0.2583 > 0.05) (Fig. S6H).

### The sTau-SA is significantly higher in PD and dementia with Lewy bodies than in multiple system atrophy and normal controls but it is still lower than that in AD

**Fig. 4:**
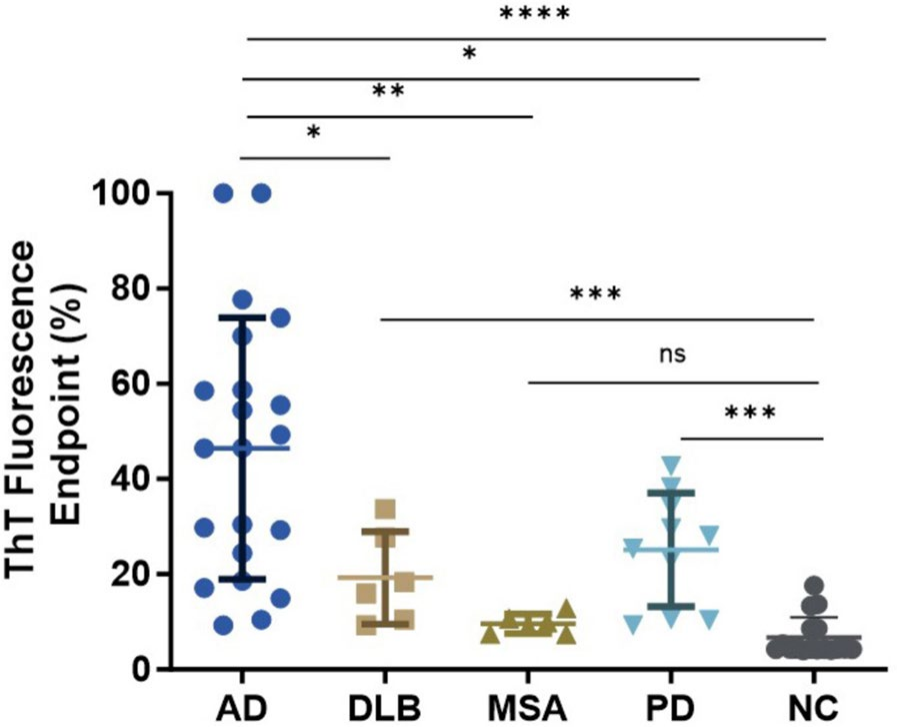
Tau-SAA of skin samples from participants with AD, synucleinopathies, and NC using 4RCF-based RT-QuIC assay. Scatter plot illustrating the distribution of tau-SA at the endpoint fluorescence readings across skin samples from 21 cases with AD, different synucleinopathies including 6 cases with DLB, 6 with MSA and 10 with PD as well as 17 NCs. *: *p* < 0.05, **: *p* < 0.01, ***: *p* < 0.001; ****: *p* < 0.0001

Accumulation of tau aggregates in the brain has been observed in some of cases with synucleinopathies including PD, dementia with Lewy bodies (DLB), and multiple system atrophy (MSA) [23–27]. Next, we further explored whether tau-SA can be detected in the skin of synucleinopathies by our sTau-SAA. Autopsy skin samples from AD (*n* = 21), PD (*n* = 10), MSA (*n* = 6), DLB (*n* = 6), and NC (*n* = 17) were examined by 4RCF-based tau-SAA. The ThT endpoint fluorescence intensity of sTau-SAA was dramatically higher in AD than in synucleinopathies, whereas the skin-tau fluorescence intensity was also significantly increased in synucleinopathies except for MSA than in the control group (Fig. 4), consistent with the previous observations that some of cases with synucleinopathies can have tau-pathology [23, 25–27]. Notably, 4 out of 6 DLB samples also had coexisting AD pathology as indicated in Table 1. In MSA samples, none of these cases had co-morbidities with confirmed tauopathies. However, tau tangles and Aβ plaques were detectable in their brain tissues (Table 1). Ten PD cadavers showed no AD pathology (Table 1) but they had significantly higher skin tau-SA compared to the normal controls (Fig. 4).

**Table 1:** Demographic and neuropathological features of autopsied cases in different groups

Neuropathological diagnosis	No. cases	Sex (M/F)	Age (years, mean ± SD)	Post-mortem Interval (hours, mean ± SD)	Braak score	Plaque total (mean ± SD)	Tangle total (mean ± SD)	Comorbidities
Alzheimer’s disease	46	18/28	76.25 ± 8.67	7.08 ± 2.93	43% IV, 13% V, 44% VI	14.1 ± 1.18	13.86 ± 2.21	4/46 with TDP-43 proteinopathy
Corticobasal degeneration	5	3/2	72.5 ± 8.20	5.74 ± 2.13	100% V	3.80 ± 5.84	12.90 ± 0.42	1/5 with AD
Progressive supranuclear palsy	33	23/10	82.73 ± 11.90	6 ± 2.77	15% III, 60% IV, 25% V	4.74 ± 5.69	7.67 ± 2.39	4/33 with Lewy bodies by Unified LB Stage
Pick’s disease	6	2/4	71.5 ± 7.66	7.5 ± 1.48	33% II, 67% III	3.33 ± 4.14	4.08 ± 3.43	1/6 with AD
Parkinson’s Disease	10	3/7	80.1 ± 6.87	4.67 ± 1.82	60% II, 30% III, 10% IV	3.98 ± 4.98	3.32 ± 1.95	None
Dementia with Lewy Bodies	6	3/3	77.17 ± 5.34	2.80 ± 1.64	17% I, 17% II, 33%IV, 33%VI	12.54 ± 2.15	8 ± 5.47	4/6 with AD
Multiple system atrophy	6	4/2	71.67 ± 7.53	3.27 ± 0.85	33% I, 17%II, 33% III, 17% IV	4.96 ± 5.71	3.08 ± 2.22	None
Non-neurodegenerative Controls	43	27/16	79.44 ± 14.19	0	37% I, 28% II, 35% III	1.44 ± 2.13	2.69 ± 1.62	None

### Biopsy sTau-SA is significantly higher in tauopathies than in normal controls

**Fig. 5:**
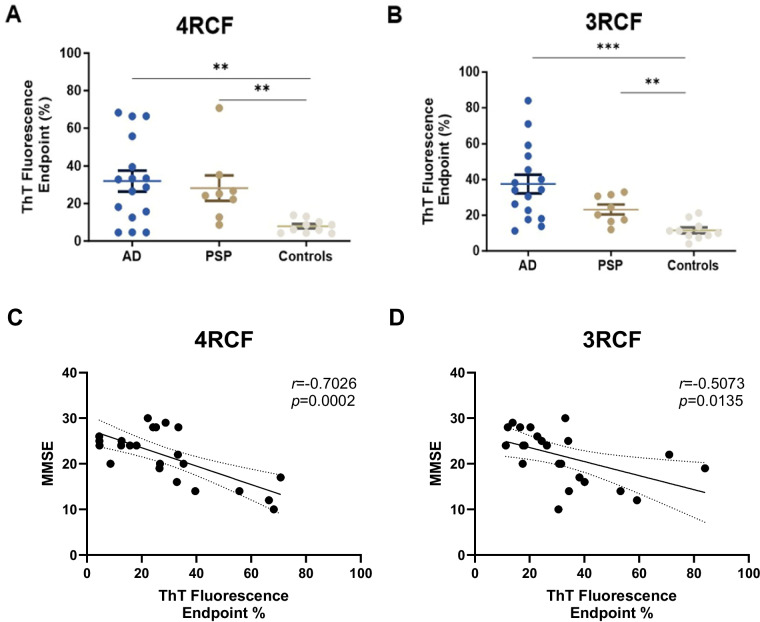
Examination of tau-SA in biopsied skin samples from patients with AD and PSP. The scatter plot displays the endpoint ThT fluorescence percentage of skin tau-SA from AD (*n* = 16), PSP (*n* = 8), and NC (*n* = 10) detected by RT-QuIC using 4RCF (**A**) and 3RCF (**B**) as the substrates. Correlation analysis between MMSE and skin tau RT-QuIC seeding activity is shown in **C** (4RCF) and **D** (3RCF) **: *p* < 0.01; ***: *p* < 0.001

We then used the above 4RCF- or 3RCF-based SAA (4RCF- or 3RCF-SAA) to examine biopsied skin samples from AD (*n* = 16), PSP (*n* = 8) and NCs (*n* = 10). Skin 4RCF-SA was significantly higher in AD than in normal controls [83,091 ± 57,912 (mean ± SD) vs. 20,490 ± 9,307, *p* = 0.0026 < 0.005]; skin 4RCF-SA was also significantly greater in PSP than in normal controls (73,360 ± 49,625 vs. 20,490 ± 9,307, *p* = 0.0043 < 0.005) (Fig. 5A). Similar to skin 4RCF-SA, 3RCF-SA was significantly higher in AD than in NCs (97,511 ± 54,115 vs. 30,090 ± 13,657, *p* = 0.0008 < 0.001) and greater in PSP than in NCs (60,449 ± 20,492 vs. 30,090 ± 13,657, *p* = 0.0017 < 0.005) (Fig. 5B). There were no significant differences in sTau-SA between AD and PSP cases with both substrates (Fig. 5). We also conducted a correlation analysis between tau seeding amplification assay (SAA) results and cognitive decline, as measured by the Mini-Mental State Examination (MMSE). Our findings indicate an inverse correlation between both 4R and 3R tau SAA and MMSE scores. Specifically, 4R tau-SA demonstrated an inverse correlation with MMSE (*r* = -0.7026, *p* = 0.002; Fig. 5C), while 3R tau-SA showed a milder, yet statistically significant, inverse correlation with MMSE (*r* = -0.5073, *p* = 0.0135; Fig. 5D). This observation implied the potential for sTau-SA to serve as an antemortem diagnostic biomarker to differentiate tauopathies from normal controls. The individual clinical data are listed in Tables 2 and 3.

**Table 2:** Demographic and clinical features of biopsied cases in different groups

Clinical diagnosis	Number of cases	Sex (M/F)	Age (yrs., mean ± SD)	Disease duration (yrs., mean ± SD)	MMSE (mean ± SD)	Hoehn & Yahr stage (mean ± SD)
Alzheimer’s disease	16	6/10	70.13 ± 9.68	3.04 ± 2.14	19.35 ± 5.44	0
Progressive supranuclear palsy	8	5/3	75.71 ± 9.36	3.5 ± 1.93	25 ± 4.38	3.27 ± 1.44
Non-neurodegenerative disease	10	3/7	68.80 ± 10.83	0	Not available	0

**Table 3:** Demographic and clinical features of individual biopsied cases in different groups

Clinical diagnosis	Age	Sex	Disease duration (years)*	Hoehn & Yahr stage	MMSE*	MoCA*	Cohort	Biopsy position
AD1	71	F	3	0	19	N/A	Italy	lateral to C7
AD2	81	M	2	0	22	N/A	Italy	lateral to C7
AD3	80	M	1	0	10	N/A	Italy	lateral to C7
AD4	78	F	4	0	14	N/A	Italy	lateral to C7
AD5	65	M	3	0	12	N/A	Italy	lateral to C7
AD6	55	M	5	0	24	N/A	Italy	lateral to C7
AD7	54	M	2	0	26	N/A	Italy	lateral to C7
AD8	61	M	2	0	24	N/A	Italy	lateral to C7
AD9	81	F	1	0	25	N/A	Italy	lateral to C7
AD10	83	F	3	0	16	N/A	Italy	lateral to C7
AD11	63	F	2	0	24	N/A	Italy	lateral to C7
AD12	76	F	8	0	N/A	N/A	Italy	lateral to C7
AD13	65	F	3	0	28	N/A	Italy	lateral to C7
AD14	62	F	2	0	24	N/A	Italy	lateral to C7
AD15	71	F	7	0	17	N/A	Italy	lateral to C7
AD16	71	F	1	0	14	N/A	Italy	lateral to C7
PSP1	72	M	1	2	20	N/A	Italy	lateral to C7
PSP2	65	F	3	2	28	N/A	Italy	lateral to C7
PSP3	59	F	2	1	29	N/A	Italy	lateral to C7
PSP4	88	F	2	4	20	21	Cleveland	lateral to C7
PSP5	84	M	7	5	20	25	Cleveland	lateral to C7
PSP6	75	M	4	2.5	25	15	Cleveland	lateral to C7
PSP7	76	M	5	4	30	21	Cleveland	lateral to C7
PSP8	79	M	4	4	28	28	Cleveland	lateral to C7
NC1	59	F	N/A	0	N/A	26	Cleveland	lateral to C7
NC2	69	M	N/A	0	N/A	27	Cleveland	lateral to C7
NC3	72	F	N/A	0	N/A	28	Cleveland	lateral to C7
NC4	63	F	N/A	0	N/A	26	Cleveland	lateral to C7
NC5	81	F	N/A	0	N/A	25	Cleveland	lateral to C7
NC6	71	M	N/A	0	N/A	28	Cleveland	lateral to C7
NC7	73	F	N/A	0	N/A	27	Cleveland	lateral to C7
NC8	74	F	N/A	0	N/A	25	Cleveland	lateral to C7
NC9	77	F	N/A	0	N/A	30	Cleveland	lateral to C7
NC10	77	M	N/A	0	N/A	23	Cleveland	lateral to C7

* *N/A* Not applicable

### ThT fluorescence levels of sTau-SAA end-point correlate with dot-blot intensity of captured tau aggregates of the RT-QuIC end products

**Fig. 6:**
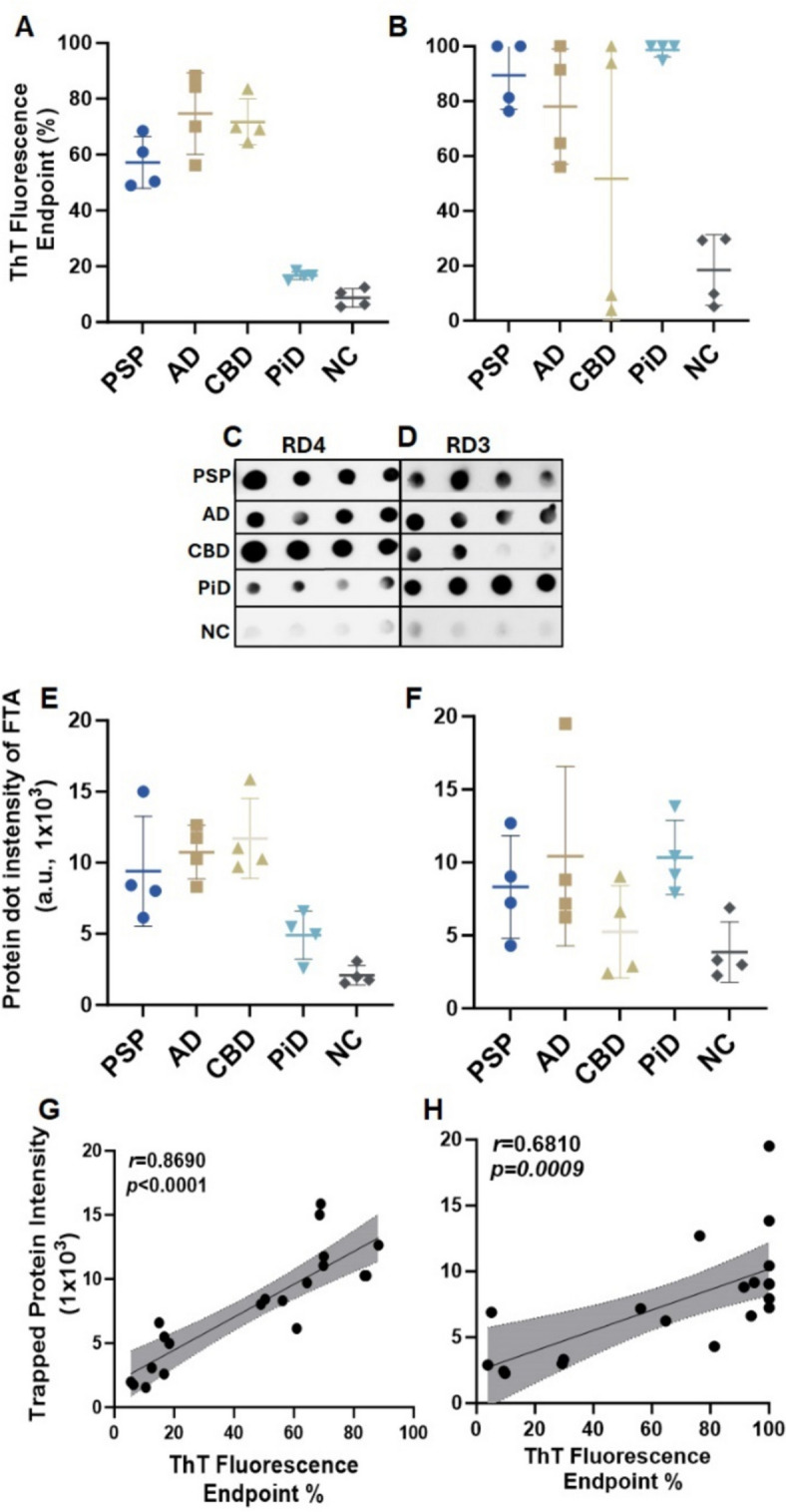
Characterization of RT-QuIC end products of skin tau from tauopathies using filter-trap assay probed with anti-3R (RD3) and anti-4R (RD4) tau antibodies. Scatter plots of ThT fluorescence values skin-tau RT-QuIC end products of selected samples from PSP (*n* = 4), AD (*n* = 4), CBD (*n* = 4), PiD (*n* = 4) and NC (*n* = 4), with 4RCF (**A**) and 3RCF(**B**) as the substrates. The filter-trap assay (FTA) of 3RCF-/4RCF-based RT-QuIC end products of skin samples from different tauopathies including PSP, AD, CBD, PiD and NC (4 cases/group) probed with RD4 (**C**) or RD3 (**D**) antibodies. Quantitative densitometry of FTA-dot blotting with 4RCF- (**E**) and 3RCF (**F**) -based RT-QuIC end products from panels (**C**) and (**D**). Correlation analysis between FTA-trapped protein dot intensity and skin tau-SA of 4RCF- (**G**)/3RCF(**H**) -based RT-QuIC end products

To determine whether ThT fluorescence levels reflecting sTau-SA represent the formation of skin tau-seeded aggregates, we correlated the end-point ThT fluorescence levels with the dot-blot intensity of tau aggregates captured by a filter-trap assay (FTA) (Fig. 6). After obtaining the end point ThT fluorescence levels of sTau-SA of 4 cases each from AD, PSP, CBD, PiD and NC with 4RCF or 3RCF as the substrate (Fig. 6A, B), we then ran FTA with their corresponding end products, followed by probing the dot-blots with anti-4R tau antibody (RD4) (Fig. 6C) and anti-3R antibody (RD3) (Fig. 6D). The semiquantitative densitometric scanning of protein dot intensity on the dot-blots revealed that similar to ThT fluorescence levels, the intensity of tau aggregates captured by FTA on the blots was significantly higher in tauopathies than in normal controls by both RD4 (Fig. 6E) and RD3 antibodies (Fig. 6F). Correlation analyses demonstrated that the intensity of the trapped aggregates from the end products correlated positively with the ThT fluorescence levels (*r* = 0.87 for 4R, *p* < 0.001; *r* = 0.68 for 3R, *p* < 0.009) (Fig. 6G, H).

### Transmission electron microscopy of 4RCF- or 3RCF-based RT-QuIC end products displays the protofibril-like structures

**Fig. 7:**
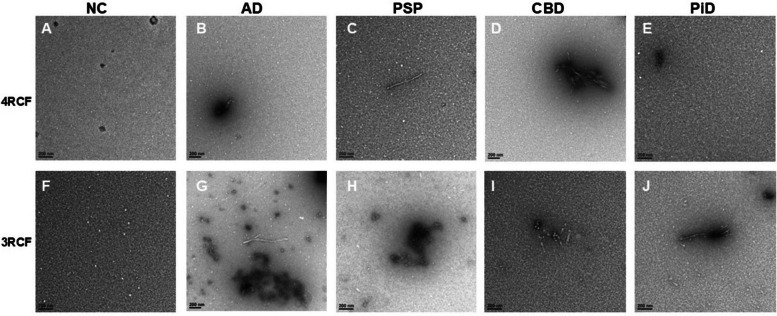
Transmission electron microscopy of SAA end products of skin misfolded tau from AD, other tauopathies and normal controls. Panels (**A**) through (**E**) show the representative images of tau 4RCF-based RT-QuIC end-products with normal controls (NC, **A**), AD (**B**), PSP (**C**), CBD (**D**) and PiD (**E**). Panels (**F**) through (**J**) exhibit the representative images of tau 3RCF-based RT-QuIC end-products with NC (**F**), AD (**G**), PSP (**H**), CBD (**I**) and PiD (**J**). Scale bars: 200 nm

While our FTA apparently was able to detect captured tau aggregates, to further determine the morphology of the amplified skin tau aggregates, we performed transmission electron microscopy (TEM) of the RT-QuIC end-products of skin misfolded tau from 3 cases each of AD, PSP, CBD, PiD and NCs with either 4RCF or 3RCF as the substrate (Fig. 7). For 4RCF, TEM revealed that except for PiD and NCs (Fig. 7A, E), other end-products had a small number of protofibril-like structures. For 3RCF, while NCs showed only oligomer-like structure (Fig. 7F), protofibrils were detectable in the end-products of tau RT-QuIC of cases with all tauopathies by TEM (Fig. 7G and J).

### The end-products of 4RCF- and 3RCF-SAA exhibit different patterns of resistance to proteinase K digestion

**Fig. 8:**
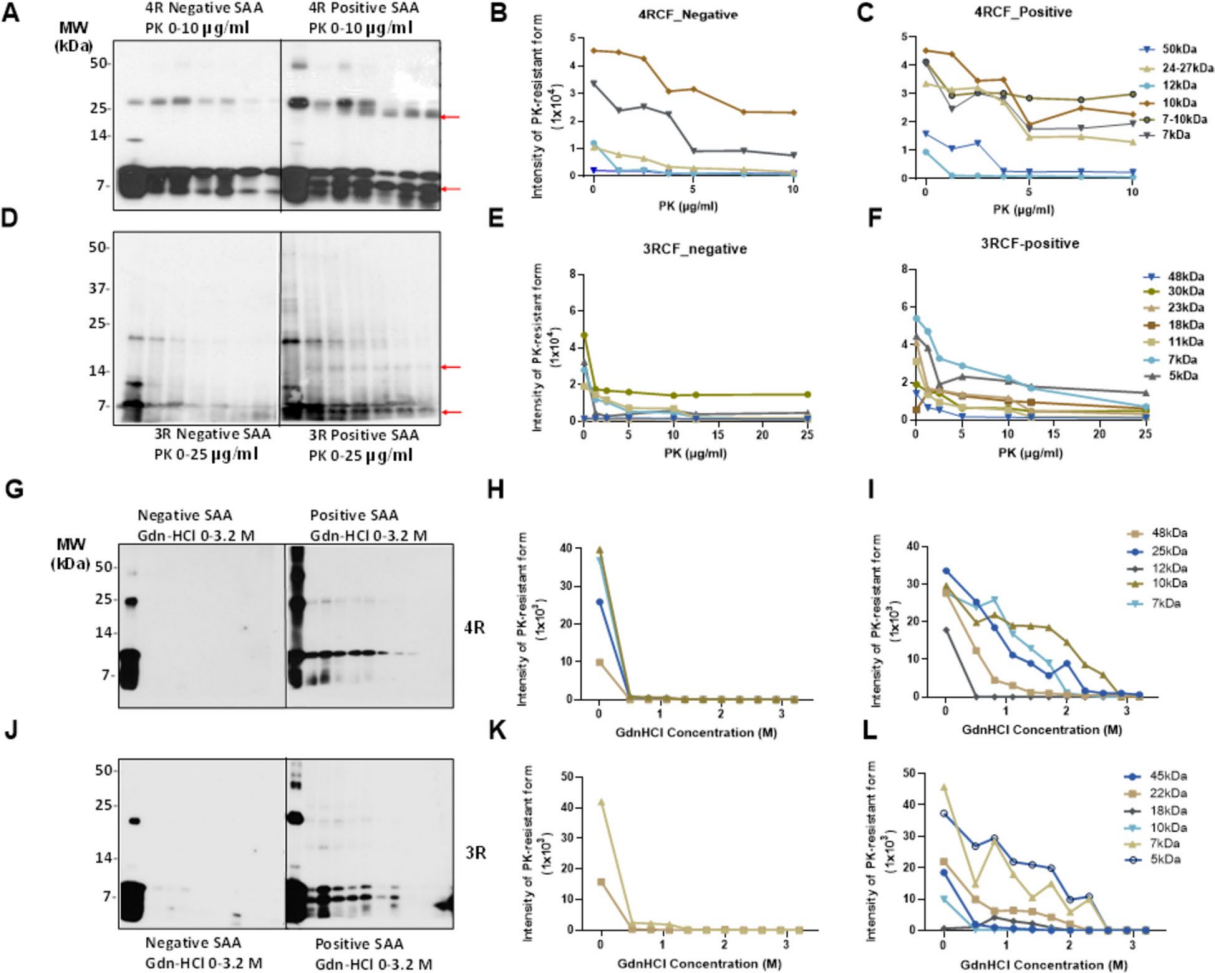
PK-resistance and conformational stability assay of the end products of 4RCF-/3RCF-based tau RT-QuIC assay of skin samples from AD and controls.** A-C** Western blotting of PK titration of 4RCF-based SAA end-products of AD and non-AD skin samples and quantitative analysis of intensity of PK-resistant tau fragments with different molecular weights by densitometry. **D**-**F**: Western blotting of PK titration of 3RCF-based SAA end products of AD and non-AD skin samples and quantitative analysis of intensity of PK-resistant tau fragments with different molecular weights by densitometry. Probed with RD3 and RD4 antibodies against 3R or 4R tau isoforms, respectively. The 4RCF-(**G**)/3RCF(**J**)-based RT-QuIC end products of AD and non-AD skin samples were treated with different concentrations of GdnHCl and followed by PK digestion prior to western blotting probed with anti-tau antibodies RD4 against 4R (**G**) and RD3 against 3R (**J**) tau fragments. Quantitative analysis of GdnHCl/PK-resistant protein band intensity with different molecular weights by densitometry on blots (**G** and **J**). **H** and I for 4R tau; **K** and **L**: for 3R tau

The pattern of protein aggregates from the skin tau RT-QuIC end-products to proteinase K (PK) digestion has been widely believed to reflect the conformational properties of misfolded proteins examined. Since the small fragments of 3RCF tau lower than 7 kDa were more difficult to digest by PK, we next performed the titration of varied PK concentrations ranging from 0, 1.25 µg/mL, 2.5 µg/mL, 3.75 µg/mL, 5 µg/mL, 7.5 µg/mL, to 10 µg/mL for the 4R (Fig. 8A) and 0, 1.25 µg/mL, 5 µg/mL, 10 µg/mL, 12.5 µg/mL and 25 µg/ml for the 3R tau (Fig. 8B) SAA end-products of skin tau from AD and non-AD subjects. Without PK treatment, the end-products of 4RCF-based RT-QuIC of skin tau from non-AD exhibited 3 protein bands by the RD4 tau antibody, migrating at approximately 25–26 kDa, 12–14 kDa, and 7–10 kDa (Fig. 8A). The short exposure of our blots showed that the 7–10 kDa bands actually consisted of 7 kDa and 10 kDa proteins. As a result, the above four bands could represent the trimer and dimer of a 7 kDa band as well as the monomers of a full-length 4RCF (~ 12 kDa) and a truncated 4RCF (~ 7–10 kDa), respectively. Of them, the two lower monomeric bands were predominant, whereas the top dimeric and trimeric tau bands were underrepresented, accounting for less than 1–2% of total tau (Fig. 8A, C). In contrast, the end-product from AD skin samples without PK-treatment exhibited an additional band migrating at approximately 48–50 kDa in addition to the four bands found in the end-product of non-AD skin described above. This band could be oligomers of full-length or truncated tau molecules. In addition, the intensity of truncated trimers and dimer of tau migrating at 24–27 kDa was significantly increased compared to that of non-AD end-products.

Upon PK-treatment, for the non-AD end-products, the intensity of the two lower monomeric tau bands was significantly decreased while they were still detected at PK of 10 µg/mL (Fig. 8A, B). The intensity of the tau band migrating at ~ 25–27 kDa was increased first up to PK of 2.5 µg/mL and then decreased, until became undetectable at PK of 10 µg/mL. The band migrating at ~ 12 kDa seemed to be completely PK-sensitive and no band was detectable even at the lowest PK concentration at 1.25 µg/mL. In contrast, after PK-treatment the end-products of the RT-QuIC with AD skin samples showed the decreased intensity of the tau band migrating at ~ 25–27 kDa but generated additional smaller band migrating at about 24 kDa (Fig. 8A, C, red arrow). This band was most likely derived from the truncation of the 25–27 kDa band since it was generated and enhanced over the increase in the PK concentration. In contrast with the non-AD end-products, AD end-products also exhibited an additional band between 7 kDa and 10 kDa migrating at about 8 kDa in the PK-treated AD skin en- products (marked with the red arrow in Fig. 8A). The intensity of this band was similar to that of 7 kDa and 10 kDa bands, and showed no changes upon the increase in the PK concentration (Fig. 8A, C).

Regarding the end-products of sTau-SAA using 3RCF as the substrate, without PK-treatment, the AD and non-AD samples all mainly exhibited 3 bands migrating at about 7 kDa, 10–11 kDa, and 22–23 kDa on the gels (Fig. 8D). According to the sequence of the 3RCF molecule, the molecular weight of the monomeric 3RCF should be 10.5 kDa. Therefore, the 7 kDa band could be a truncated fragment of 3RCF while 22–23 kDa band could be a dimer of 3RCF. Since we got high intensity of the low molecular weight band (~ 7 kDa), we increased the PK concentration to 25 µg/mL for 3R and decreased the loading amounts of samples (Fig. 8D, E). The intensity of the 3 bands from non-AD end-products all decreased while there was a faint band emerging, migrating at approximately 5 kDa over the increase in PK concentrations. The intensity of the 3 bands from AD skin tau RT-QuIC end-products was also all decreased while there were two additional bands emerging, migrating at approximately 16–18 kDa and 5–6 kDa over the increase in PK concentrations (Fig. 8D, F, red arrows). The monomers of truncated 4RCF (at ~ 12 kDa) and 3RCF (at ~ 10–11 kDa) exhibited no resistance to PK treatment (Fig. 8A, D). However, the dimers at higher molecular weights demonstrated increased resistance to PK, with the exception of the 4R negative end-product dimers, which were digested at PK concentrations exceeding 7.5 µg/mL. The low molecular weight bands below the monomers were more resistant to being digested within the chosen PK concentration range, especially with 4RCF.

### Conformational-stability assay of skin tau aggregates amplified by tau-SAA

We treated 4RCF (Fig. 8G) and 3RCF (Fig. 8J) tau-SAA end-products with GdnHCl ranging from 0 to 3.2 M, followed by PK digestion at 10 µg/mL and quantitative analyses of GdnHCl/PK-resistant protein intensity of each treated sample. This approach is grounded on the principle that subtle differences in protein structure can be ascertained by assessing conformational stability when the protein is exposed to a denaturant such as GdnHCl at appropriate concentration ranges [28]. In the absence of GdnHCl and PK, 3 tau bands migrating at 48 kDa, 25 kDa and 7–12 kDa were observed for 4RCF-based RT-QuIC end-products, while 2 bands migrating at 22–23 kDa and 5–10 kDa were detected in 3RCF-based RT-QuIC end-products (Fig. 8G, J). In contrast, both AD skin 4RCF-/3RCF-based tau RT-QuIC end-products were found to have multiple or smear bands above 30 kDa (Fig. 8G, J). After GdnHCl and PK-treatment, there were virtually no PK-resistant tau bands detectable from both non-AD 4RCF/3RCF-based RT-QuIC end products. In contrast, positive skin tau RT-QuIC from AD participants with either 4RCF or 3RCF as the substrate showed PK-resistant tau fragments, especially for bands migrating at 25 kDa or lower for low concentration of GdnHCl (Fig. 8G through L). Notably, there was an additional partially PK-resistant tau fragment migrating between 7 kDa and 5 kDa bands for 3RCF-based RT-QuIC end-products (Fig. 8J, L), which was not detectable in the 4RCF-based skin tau RT-QuIC end-products (Fig. 8G, I). The GdnHCl concentrations required to make half of the tau end-product sensitive to PK, referred to as GdnHCl1/2, were 2.6 M for positive 4R tau and 2.3 M for positive 3R tau for bands migrating at 22–25 kDa and 10 kDa, indicating that the positive 4R tau end-products were approximately 1.13-fold more stable than the 3R tau end product. But, the 3RCF-based RT-QuIC end products from AD cases generated stable 7 kDa band (Fig. 8J, L).

## Discussion

The demand for early, reliable, affordable and less invasive biomarkers in AD clinical practice has remained unmet. These biomarkers are essential not only for diagnosing and predicting the disease but also for facilitating patient participation in clinical trials and monitoring the effectiveness of therapeutic interventions [29]. With the recent positive outcomes from the Clarity AD trial [30] and the critical juncture in developing new strategies to prevent and slow down AD progression [31], the urgency for such biomarkers has intensified.

The recently developed AD-specific biomarkers utilized in clinical research have significantly enhanced our ability to diagnose and monitor AD pathology in living patients. These biomarkers have also provided valuable insights into the accumulation and spread of misfolded Aβ and tau aggregates in AD [32]. In both the initial 2018 and updated 2023 A/T/N research frameworks by the National Institute on Aging and the Alzheimer’s Association (NIA-AA), the detection of misfolded proteins, including Aβ and tau, through brain molecular imaging and body fluid analysis, has been central [33, 34]. A recent study assessing various brain imaging modalities in monitoring cognition and predicting cognitive decline has highlighted the significance of neocortical tau pathology as a key factor in cognitive decline over time, with tau-PET showing superior prognostic value compared to other neuroimaging measures [35]. An important advancement in the updated 2023 research framework is the inclusion of recently developed plasma biomarkers, supplementing the previously relied-upon biomarkers from CSF and brain imaging [33, 34]. However, despite these developments, there remain limitations associated with brain imaging, CSF, and Simoa-based plasma biomarkers, as outlined in the 2023 update [34]. It is also uncertain whether current biomarkers cover all relevant neuropathologies and fully reflect all aspects of pathogenesis. Moreover, the current list of AD biomarkers in the updated framework lacks markers capable of reflecting the pathological functions of misfolded proteins, such as the seeding activity of pathogenic tau or Aβ, as seen in the αSyn-SAA for PD and PrP^Sc^-SAA for PrD. As a result, there is an ongoing quest for new minimally invasive or non-invasive biomarkers that can directly capture the unique pathogenic features of neurotoxic misfolded proteins.

Utilizing ultrasensitive RT-QuIC and/or PMCA techniques, our prior investigations have successfully identified minute quantities of misfolded proteins in skin samples obtained from individuals with PrD and PD by detecting their seeding activity, a prion-like characteristic of misfolded proteins. For example, we have illustrated that the seeding activity of PrP^Sc^ and pathogenic αSyn can be discerned in patients with Creutzfeldt-Jakob Disease (CJD), prion-infected rodents, and individuals affected by PD or other synucleinopathies, respectively [8, 36–40]. These findings have been corroborated by independent research groups [41–45]. Motivated by these discoveries, we have expanded our inquiry to encompass the most prevalent neurodegenerative condition, AD, and other tauopathies.

Our current study has unveiled several groundbreaking discoveries. First, similar to tau levels in AD brains [16, 19, 20], autopsy skin tau-SA detected by RT-QuIC was markedly higher in tauopathies than in normal controls, suggesting the potential use of sTau-SA as a novel diagnostic biomarker for tauopathies. Second, sTau-SA was detectable in cases with PD and DLB but not in MSA, although their seeding activity was significantly lower than that of AD and higher than normal controls. Third, autopsy skin tau aggregates from all tauopathies could seed the 3RCF substrate, whereas the 4RCF substrate could be seeded by skin tau aggregates from AD, PSP, and CBD but not from PiD. Fourth, the levels of skin tau-SA appeared to be associated with the progression of Braak staging observed in the brain. Fifth, biopsy skin tissues from individuals with AD and PSP showed significantly higher levels of tau-SA compared to normal controls, implying the potential of sTau-SA as a diagnostic biomarker for living tauopathy patients. Sixth, analysis of RT-QuIC end-products revealed that sTau-SAA with AD skin samples formed tau oligomers and aggregates, confirmed by FTA, PK-treatment, conformational-stability assays, and TEM. ThT fluorescence intensity at the endpoint of the reaction correlated well with tau aggregate dot intensity by FTA. Seventh, PK-treatment of skin tau RT-QuIC end-products in AD exhibited greater amounts of PK-resistant tau fragments than in normal controls. Finally, conformational-stability assays showed that skin tau could seed different strains in 4RCF and 3RCF substrates. These findings raise several important implications regarding the role of skin tau in the diagnosis and pathogenesis of tauopathies.

Studies have demonstrated an increase in tau gene expression in the skin of aging males [46]. Additionally, pathological tau deposits have been identified in peripheral organs such as the aorta, liver, spleen, and stomach of individuals with AD, but not in controls [47]. A distinct tau isoform known as big tau has been observed in peripheral tissues of both rodents and humans [48–51]. Phosphorylated tau has been detected in the skin of both AD participants and normal controls through various methods including immunohistochemistry, western blotting, and MALDI-MSI [27, 52–54]. Our study revealed that the tau-SA was lower in the skin than in the brain of AD patients. The tau-SA in both skin and brain tissues exhibited a trend of positive correlation. These findings suggest that skin tissue provides valuable insights into the levels and patterns of tau molecules, making it a promising specimen for investigating the role of tau in the pathogenesis of various tauopathies and for developing differential diagnostic tools to distinguish AD from other tauopathies and control subjects. It would be also interesting to further explore the similarity and differences in seeding activity, physicochemical features, and transmissibility between the skin and brain tau in the future.

Our study demonstrates that tau extracted from both autopsy and biopsy skin samples of individuals with AD and other non-AD tauopathies exhibits significantly higher seeding activity compared to normal controls. This suggests that sTau-SA could serve as a novel diagnostic biomarker for tauopathies. Our 4RCF- or 3RCF-based RT-QuIC assay of autopsy skin samples from AD and non-AD tauopathies achieved a sensitivity of 75–80% and a specificity of 95–100%, respectively. Tau-SA was markedly elevated in biopsy skin samples from tauopathy patients compared to controls. Furthermore, our skin tau-based RT-QuIC assay holds promise for detecting the comorbidity of AD, PD and PrD. Skin tau-, αSyn-, and PrP^Sc^-SA can be detected depending on the substrate used ([15, 38, 39, 55–57], current study). Therefore, if a case exhibits tau-, αSyn-or PrP^Sc^-pathology in the brain, our RT-QuIC assay is likely to detect tau-, αSyn-, or PrP^Sc^-SA in the skin, indicating comorbidity of AD, PD, and/PrD. However, reliable diagnosis of comorbidity requires ensuring that it does not result from cross-seeding between tau, αSyn, and PrP^Sc^ as tau-seeds may trigger αSyn or PrP aggregation and vice versa. Moreover, our findings regarding sTau-SA patterns shed light on the heterogeneity of tauopathies. Skin tau from PiD, characterized by 3R-dominated tau aggregates in the brain, seeded the 3RCF but not the 4RCF substrate in the RT-QuIC assay. In contrast, skin tau from AD, which features a mixture of 3R/4R tau, and from PSP/CBD, characterized by 4R-dominated tau aggregates in the brain, seeded both 4RCF and 3RCF substrates. These observations underscore the complexity of tauopathies and highlight the potential of skin tau-SA as a diagnostic biomarker to differentiate among different tauopathy subtypes.

Our investigation of skin-tau RT-QuIC end-products confirmed that the increased tau-SA was accompanied by the formation of tau aggregates, as evidenced by our FTA, PK treatment, and conformational stability assays. TEM revealed the presence of oligomers and protofibrils, rather than mature fibrils, in the 3RCF- or 4RCF-based RT-QuIC end products of skin tau from AD cases, consistent with findings in the existing literature [22, 58]. Our previous study demonstrated that recombinant 4RCF fragments can spontaneously form mature fibrils in vitro within approximately two weeks [20]. Furthermore, analysis of PK-digested skin tau RT-QuIC end-products showed the generation of additional PK-resistant tau fragments in positive RT-QuIC end-products. Importantly, 3RCF- and 4RCF-based RT-QuIC end-products generated different PK-resistant fragments, suggesting structural differences between the two. It will be intriguing to investigate whether the end-products of tau-SAA with brain and skin samples yield similar or different structural and physicochemical features in future studies. This exploration could provide valuable insights into the pathogenic mechanisms underlying tau aggregation in both the central nervous system and peripheral tissues.

## Conclusions

In summary, our research highlights the promise of utilizing skin samples as a minimally invasive and readily accessible means for diagnosing tauopathies. Detecting phosphorylated tau and tau seeding activity in skin samples has the potential to advance novel diagnostic methods for these conditions. It is imperative to conduct additional studies involving larger patient groups and refine detection techniques to fully ascertain the clinical value of a skin-based diagnostic approach for distinguishing between various tauopathies.

## Methods

### Design of the study

Skin samples were collected from two primary sources: (1) Cadavers with neuropathologically confirmed diagnoses of tauopathies and normal controls, and (2) Living patients with clinical diagnoses of AD and PSP. We performed RT-QuIC technique to examine the prion-like seeding activity of pathological tau. Statistical analyses were conducted to assess the sensitivity and specificity of the assay, utilizing different tau substrates to understand the impact on assay performance. All cadaveric skin samples were collected post-mortem and stored according to established protocols to preserve the biochemical properties of the tissue. Living patient samples were obtained through skin biopsies performed in a clinical setting and were immediately transported to the laboratory for analysis.

### Ethical statement

All procedures and protocols were monitored and approved by the Institutional Review Boards (IRBs) of University Hospitals Cleveland Medical Center, Banner Sun Health Research Institute, and IRCCS Institute of Neurological Sciences of Bologna. Written informed consent was obtained from all living subjects undergoing skin biopsy or from family members for skin autopsy. For post-mortem sample collection, we obtained the specimens with respect to the wishes of the deceased individuals and their families, following all legal and ethical guidelines. For skin biopsy procedures, all participants provided their informed consent prior to their inclusion in the study.

### Reagents and antibodies

Proteinase K (PK) and guanidine hydrochloride (GdnHCl) were purchased from Sigma Chemical Co. (St. Louis, MO, USA). Reagents for enhanced chemiluminescence (ECL Plus) were from Amersham Pharmacia Biotech, Inc. (Piscataway, NJ). Anti-tau mouse monoclonal antibodies RD3 and RD4 (Sigma-Aldrich) against human tau repeating region and sheep anti-mouse (SVM) IgG conjugated with horseradish peroxidase as a secondary antibody (AC111P, CHEMICON International, Inc, Burlington, MD) were used. Antibodies against Phospho-Tau (Thr231) and phospho-Tau (Ser396) were purchased from Cell Signaling Technology (Danvers, MA).

#### Source of skin samples

A total of 135 autopsy scalp skin samples from AD (*n* = 46), PSP (*n* = 33), CBD (*n* = 5), PiD (*n* = 6) and non-neurodegenerative controls (NNCs, *n* = 46) were collected and examined. These samples were obtained from the Arizona Study of Aging and Neurodegenerative Disorders (ASAND)/Brain and Body Donation Program at Banner Sun Health Research Institute through the Biomarkers across Neurodegenerative Diseases Research Grant 2019 (BAND 3) study. The diagnoses of these cases were confirmed via neuropathological examination of autopsied brain tissues at the ASAND. Biopsied skin samples from C7 paravertebral site (5 cm from the midline) of clinically diagnosed AD (*n* = 16), PSP (*n* = 8) and normal controls (*n* = 10) were from the Bellaria Hospital, Bologna, Italy, and the University Hospitals Cleveland Medical Center, Cleveland, Ohio, USA (see neuropathological and clinical information in Tables 1 and 2).

### Plasmid constructs cloning

Expression vectors for all six full-length wild-types human tau isoforms were generously provided by Dr. George Bloom of the University of Virginia (originated from the late Dr. Lester “Skip” Binder and Dr. Nicolas Kanaan of Michigan State University) [20, 21]. 3RCF construct (three microtubule-binding repeats and cysteine-free construct containing C322S mutation) was first PCR-amplified of 3R repeats sequence from 2N3R tau plasmid and cloned into the same expression vector using Nde I and Xho I restriction sites, followed by site-directed mutagenesis at Cys322 site to Serine using QuikChange Site-directed mutagenesis kit (Agilent, Santa Clara, CA). 4RCF construct (four microtubule-binding repeats and cysteine-free construct containing C291S and C322S mutations) was first PCR-amplified of 4R repeats sequence from 2N4R tau plasmid and cloned into the same expression vector using Nde I and Xho I restriction sites, followed by site-directed mutagenesis at Cys291 and Cys322 sites using QuikChange Site-directed mutagenesis kit. All constructs were designed with a his6-tag at their carboxy-termini to facilitate protein purification and were verified by DNA sequencing.

### Engineered tau fragments 3RCF and 4RCF expression and purification

Recombinant 3RCF and 4RCF was prepared as previously described [20]. In brief, plasmids encoding human tau engineered constructs 3RCF and 4RCF were transformed into BL21-DE3 E. coli cells. Overnight starter cultures of BL21-DE3 E. coli cells transformed with recombinant tau plasmids were inoculated into multi-liter LB broth at 1:50 dilution and 100 mg/mL ampicillin. Cultures were incubated at 37 °C, shaking until OD600 reached between 0.5 and 0.6. Tau expression was induced using 1 mM IPTG and continued to grow for an additional 4 h. BL21-DE3 cells containing expressed tau were pelleted and resuspended in 50 mM NaH2PO4, pH 8.0 and 300 mM NaCl (sonication lysis buffer) at a concentration of 20 mL/L of culture preparation and sonicated at 60% power in ten 30-second intervals over 10 min. Cell lysates were centrifuged and supernatant containing the protein was applied to Ni-NTA column equilibrated with sonication lysis buffer. The columns were washed with 40–50 times of bed volumes of column buffer (sonication lysis buffer) followed by washing buffer (50 mM NaH2PO4, pH 8, 300 mM NaCl, and 20 mM imidazole). Recombinant protein was then eluted using elution buffer (50 mM NaH2PO4, pH 8, 300 mM NaCl, and 200 mM imidazole). Fractions were tested for protein concentration using 5 µL of protein sample mixed with 10 µL Coomassie Protein Assay reagent (ThermoFisher Scientific). Pooled fractions were concentrated to 4 mL using 10 kDa molecular weight cut-off spin columns (Millipore) and filtered using 0.22 μm low-binding Durapore PVDF membrane filters (Millipore). 3RCF and 4RCF tau proteins were further purified by FPLC using size exclusion Superdex-75 and Superdex-200 columns (GE Healthcare) in 1 x PNE buffer (25 mM PIPES, 150 mM NaCl and 1 mM EDTA, pH 7.0). Final 3RCF and 4RCF proteins were over 90% purity as evaluated by SDS-PAGE. Protein concentrations were quantified by BCA protein assays (ThermoFisher Scientific).

### Skin tissue preparation

Skin samples of approximately 30–100 mg in weight and 3–5 mm x 3–5 mm in size, primarily contained epidermis and dermis were collected as previously and prepared described [15, 39]. Briefly, skin tissues were homogenized at a 10% (w/v) concentration in a lysis buffer containing 2 mM CaCl2 and 0.25% (w/v) collagenase A (Roche) in Tris-Buffered Saline (TBS). The samples were incubated in a shaker at 37 °C for 4 h, shaking at 500 rpm, followed by homogenization using a Mini-BeadBeater (BioSpec, Laboratory Supply Network, Inc., Atkinson, NH).

### RT-QuIC analysis

The RT-QuIC assay was modified as previously described with a slight modification [15, 18–20, 59]. In brief, the reaction mix for skin tau was prepared with 10 mM HEPES, pH 7.4, 200 mM NaCl, 10 µM ThT, and 10 µM either 4RCF or 3RCF tau substrate. In a 96-well plate (Nunc), 98 µL aliquots of the reaction mix were added to each well, followed by seeding with 2 µL of diluted skin homogenate (1:200 from 5% homogenate supernatant prepared by centrifugation at 3,000 g for 10 min at 4 °C) in 10 mM HEPES, 1 x N2 supplement (Gibco), 1 x PBS and centrifuged at 5,000 g for 5 min at 4 °C. The N2 supplement was used for reducing non-specific seeding activity. The plate was sealed with a plate sealer film (Nalgene Nunc International) and then incubated at 37 °C in a BMG FLUOstar Omega plate reader. The incubation involved cycles of 1 min of orbital shaking followed by a 15-min of rest for the specified duration. ThT fluorescence measurements from bottom read (450 ± 10 nm excitation and 480 ± 10 nm emission) were recorded every 45 min. Gain was set at 1800–2000, varied from different readers with thebottom reading. Each sample dilution contained 4 replicate reactions. The average ThT fluorescence values per sample were calculated using data from all four replicate wells, regardless of whether they crossed the threshold defined by ROC. A sample was considered positive if at least 2 of 4 replicate wells exceeded this threshold.

To quantify tau-SA detected by RT-QuIC, end-point dilution titrations were employed to determine the estimates of the sample dilution that generated positive reactions in 50% of the replicate reactions as the 50% seeding dose or SD50 (usually 2 out of 4 replicates) [15].

### RT-QuIC assay of AD skin tau with or without immunodepletion by anti-tau antibody

The skin homogenates from AD were centrifuged at 5,000 g for 5 min at 4 °C and the pellets were discard. A 100 µl of immunoprecipitation (IP) lysis buffer containing 1% BSA, 150 mM NaCl, 1% Triton X-100, 50 mM Tris, pH 7, and protease inhibitor was mixed with 1 µg of Tau5 antibody directed against total tau. The mixture was incubated with 30 µL of A/G magnetic beads (Pierce, MA) at 4 °C, ensuring rotation overnight. A 100 µl of the supernatant from the skin homogenates was incorporated and rotated at 4 °C for 4 h. Then, a magnetic rack was used to separate and retain the supernatant that was used as seeds in subsequent Immunodepletion RT-QuIC procedures.

### Conformational stability immunoassay

The conformational stability immunoassay of RT-QuIC end products was conducted as previously described with a minor modification [28]. Briefly, 20 µL aliquots of end products were mixed with 20 µL of GdnHCl stock solution, resulting in final GdnHCl concentrations ranging from 0 to 3.0 M. After incubating at room temperature for 1.5 h, samples were precipitated with a 5-fold volume excess of pre-chilled methanol overnight at -20 °C. Following centrifugation at 14,000 g for 30 min at 4 °C, the pellets were resuspended in 20 µL of lysis buffer (10 mM Tris-HCl, 150 mM NaCl, 0.5% Nonidet P-40, 0.5% deoxycholate, 5 mM EDTA, pH 7.4). Each aliquot was digested with 10 µg/mL PK for 30 min at 37 °C. The reaction was terminated with cOmplete protease inhibitor cocktail (CO-RO, Roche), and the samples were boiled in SDS loading buffer and loaded onto 15% Tris-HCl pre-cast gels (Bio-Rad) for Western blotting analysis.

### Western blotting

The samples prepared as described above were separated using 15% Tris-HCl Criterion pre-cast gels (Bio-Rad) in SDS-PAGE. Proteins from the gels were transferred onto Immobilon-P polyvinylidene fluoride (PVDF, Millipore) membranes for 90 min at 70 V. To probe with the anti-tau antibodies (RD3, RD4, pT231, or pS396), the membranes were incubated overnight at 4 °C with a 1:1,000–1:4,000 dilution of the primary antibodies. After incubation with a 1:4,000–1:5,000 dilution of horseradish peroxidase-conjugated sheep anti-mouse IgG, tau bands were visualized on Kodak film using ECL Plus as instructed by the manufacturer. Densitometric analysis was used to measure the intensity of tau protein bands, which were quantified with UN-SCAN-IT Graph Digitizer software (Silk Scientific, Inc., Orem, Utah).

### Filter-trap assay

The filter-trap assay was used to determine the RT-QuIC reaction mixtures with increased ThT fluorescence formed aggregates and to evaluate their sizes as described previously [60]. In brief, the end products of RT-QuIC were mixed with washing buffer containing 2% SDS, 10 mM Tris-HCl, pH 8.0 and 150 mM NaCl for an hour at room temperature. Following incubation, the samples were filtered through a cellulose acetate membrane (Advantec MFS, Dublin, CA). After filtering, the membrane was rinsed with washing buffer to remove unbound proteins and subsequently blocked with 5% BSA in 0.1% Tween-20 in 1 x PBS for an hour. The membrane was then probed with RD3 and RD4 antibodies, followed by incubation with sheep anti-mouse secondary antibody. The proteins on the membrane were visualized using ECL Plus, and the resulting signal was captured using a chemiluminescent imaging system with X-ray/automatic film processor. Densitometric analysis was used to measure the intensity of tau protein dots for the quantitative analysis as mentioned above.

### Transmission electron microscopy

Transmission electron microscopy (TEM) images were collected as previously described [20, 61]. Briefly, the skin tau-SAA end product samples at a concentration of 10 µM were maintained in a frozen state until ready for TEM analysis. Before imaging, 2 µL of the sample was applied to a 200 mesh formvar-carbon coated grid and allowed to sit for 5 min. The excess sample was gently removed using filter paper. A thorough examination of each grid was conducted to qualitatively assess the presence of oligomers or fibrils. Representative images were taken from 15 to 20 distinct locations on each grid. The TEM studies were conducted using a JEOL-1400 transmission electron microscope (JOEL United States, Inc., Peabody, MA) at an operating voltage of 120 kV.

### Statistical analysis

Experimental data were analyzed using Student’s t-test for comparing two groups. McNemar’s test was employed to assess marginal homogeneity and differences in agreement. For comparisons between PD versus CBD and PSP, where the sample size allowed, we conducted a paired area under the ROC curve (AUC) analysis to evaluate significant differences in AUC values. Tests adopted a two-sided type II error level of 0.05.

## Supplementary Information

Supplementary Material 1.

## Data Availability

All data are available in the main text or the supplementary materials.

## Abbreviations

αSyn: α-synuclein
AD: Alzheimer's disease
CJD: Creutzfeldt-Jakob disease
CSF: Cerebrospinal fluid
CBD: Corticobasal degeneration
DLB: Dementia with Lewy bodies
FTA: Filter-trap assay
GdnHCl: Filter-trap assay
HMWB: High molecular weight band
PrP^Sc^: Infectious prion protein
MSA: Multiple system atrophy
NIA-AA: National Institute on Aging and the Alzheimer’s Association
PD: Parkinson's Disease
PiD: Pick's disease
PrD: Prion disease
PK: Proteinase K
PMCA: Protein misfolding cyclic amplification assay
PSP: Progressive supranuclear palsy
RT-QuIC: Real-time quaking-induced conversion
SAA: Seed-amplification assay
SA: Seeding activity
SVM: Seeding activity
Simoa: Single molecular immunoassay
TEM: Transmission electron microscopy

## References

[1] Coughlin D, Irwin DJ. Emerging Diagnostic and therapeutic strategies for tauopathies. Curr Neurol Neurosci Rep. 2017;17:72. 28785992 10.1007/s11910-017-0779-1 PMC5756477

[2] Prusiner SB. Biology and genetics of prions causing neurodegeneration. Annu Rev Genet. 2013;47:601–23. 24274755 10.1146/annurev-genet-110711-155524 PMC4010318

[3] Goedert M, Spillantini MG, Jakes R, Rutherford D, Crowther RA. Multiple isoforms of human microtubule-associated protein tau: sequences and localization in neurofibrillary tangles of Alzheimer’s disease. Neuron. 1989;3:519–26. 2484340 10.1016/0896-6273(89)90210-9

[4] Liu L, Drouet V, Wu JW, Witter MP, Small SA, Clelland C, et al. Trans-synaptic spread of tau pathology in vivo. PLoS One. 2012;7:e31302. 22312444 10.1371/journal.pone.0031302 PMC3270029

[5] Zhang K, Mizuma H, Zhang X, Takahashi K, Jin C, Song F, et al. PET imaging of neural activity, β-amyloid, and tau in normal brain aging. Eur J Nucl Med Mol Imaging. 2021;48:3859–71. 33674892 10.1007/s00259-021-05230-5

[6] Moda F, Pritzkow S, Soto C. Protein Misfolding Cyclic Amplification. In: Zou W-Q, Gambetti P, editors. Prions Dis. Cham: Springer International Publishing; 2023. p. 637–52. 10.1007/978-3-031-20565-1_31. [cited 2023 Aug 21].

[7] Orrù CD, Isiofia O, Hughson AG, Caughey B. Real-Time Quaking-Induced Conversion (QuIC) Assays for the Detection and Diagnosis of Human Prion Diseases. In: Zou W-Q, Gambetti P, editors. Prions Dis. Cham: Springer International Publishing; 2023. pp. 621–35. 10.1007/978-3-031-20565-1_30. [cited 2023 Aug 21].

[8] Zou W-Q, Wang Z. Seeding Activity of Skin Misfolded Proteins as a Biomarker in Prion and Prion-Like Diseases. In: Zou W-Q, Gambetti P, editors. Prions Dis. Cham: Springer International Publishing; 2023. p. 653–73. 10.1007/978-3-031-20565-1_32. [cited 2023 Aug 21].

[9] Atarashi R, Satoh K, Sano K, Fuse T, Yamaguchi N, Ishibashi D, et al. Ultrasensitive human prion detection in cerebrospinal fluid by real-time quaking-induced conversion. Nat Med. 2011;17:175–8. 21278748 10.1038/nm.2294

[10] Fairfoul G, McGuire LI, Pal S, Ironside JW, Neumann J, Christie S, et al. Alpha-synuclein RT-QuIC in the CSF of patients with alpha-synucleinopathies. Ann Clin Transl Neurol. 2016;3:812–8. 27752516 10.1002/acn3.338 PMC5048391

[11] Groveman BR, Orrù CD, Hughson AG, Raymond LD, Zanusso G, Ghetti B, et al. Rapid and ultra-sensitive quantitation of disease-associated α-synuclein seeds in brain and cerebrospinal fluid by αSyn RT-QuIC. Acta Neuropathol Commun. 2018;6:7. 29422107 10.1186/s40478-018-0508-2 PMC5806364

[12] Orrù CD, Groveman BR, Hughson AG, Manca M, Raymond LD, Raymond GJ, et al. RT-QuIC assays for Prion Disease Detection and Diagnostics. Methods Mol Biol Clifton NJ. 2017;1658:185–203. 10.1007/978-1-4939-7244-9_14 28861791

[13] Orrù CD, Groveman BR, Raymond LD, Hughson AG, Nonno R, Zou W, et al. Bank Vole prion protein as an apparently universal substrate for RT-QuIC-Based detection and discrimination of prion strains. PLoS Pathog. 2015;11:e1004983M. 26086786 10.1371/journal.ppat.1004983 PMC4472236

[14] Shahnawaz T, Tokuda M, Waragai N, Mendez R, Ishii C, Trenkwalder B, Mollenhauer C, Soto. Development of a biochemical diagnosis of Parkinson Disease by detection of α-Synuclein misfolded aggregates in Cerebrospinal Fluid. JAMA Neurol. 2017;74:163–72. 27918765 10.1001/jamaneurol.2016.4547

[15] Wang Z, Becker K, Donadio V, Siedlak S, Yuan J, Rezaee M, et al. Skin α-Synuclein aggregation seeding activity as a Novel Biomarker for Parkinson Disease. JAMA Neurol. 2020;78:1–11. 32986090 10.1001/jamaneurol.2020.3311 PMC7522783

[16] Kraus A, Saijo E, Metrick MA, Newell K, Sigurdson CJ, Zanusso G, et al. Seeding selectivity and ultrasensitive detection of tau aggregate conformers of Alzheimer disease. Acta Neuropathol (Berl). 2019;137:585–98. 30570675 10.1007/s00401-018-1947-3 PMC6426988

[17] Manca M, Standke HG, Browne DF, Huntley ML, Thomas OR, Orrú CD, et al. Tau seeds occur before earliest Alzheimer’s changes and are prevalent across neurodegenerative diseases. Acta Neuropathol (Berl). 2023;146:31–50. 37154939 10.1007/s00401-023-02574-0 PMC10261243

[18] Saijo E, Ghetti B, Zanusso G, Oblak A, Furman JL, Diamond MI, et al. Ultrasensitive and selective detection of 3-repeat tau seeding activity in pick disease brain and cerebrospinal fluid. Acta Neuropathol (Berl). 2017;133:751–65. 28293793 10.1007/s00401-017-1692-z

[19] Saijo E, Groveman BR, Kraus A, Metrick M, Orrù CD, Hughson AG, et al. Ultrasensitive RT-QuIC seed amplification assays for Disease-Associated tau, α-Synuclein, and Prion aggregates. Methods Mol Biol Clifton NJ. 2019;1873:19–37. 10.1007/978-1-4939-8820-4_2 30341601

[20] Wu L, Wang Z, Lad S, Gilyazova N, Dougharty DT, Marcus M, et al. Selective detection of misfolded tau from Postmortem Alzheimer’s Disease brains. Front Aging Neurosci. 2022;14: 945875. 35936779 10.3389/fnagi.2022.945875 PMC9352240

[21] Becker B, Brinkmalm A, Burmann BM, Perkinton M, Ashton NJ, Fox NC, et al. Tau protein profiling in tauopathies: a human brain study. Mol Neurodegener. 2024;19:54–79. 39026372 10.1186/s13024-024-00741-9 PMC11264707

[22] Wu L, Madhavan SS, Tan C, Xu B. Hexameric aggregation nucleation core sequences and diversity of pathogenic tau strains. Pathog Basel Switz. 2022;11:1559. 10.3390/pathogens11121559 PMC9784471 36558893

[23] Chin KS, Churilov L, Doré V, Villemagne VL, Rowe CC, Yassi N, et al. Tau in dementia with Lewy bodies. Aust N Z J Psychiatry. 2023;58:175–82. 37264610 10.1177/00048674231177219

[24] Hall B, Mak E, Cervenka S, Aigbirhio FI, Rowe JB, O’Brien JT. In vivo tau PET imaging in dementia: pathophysiology, radiotracer quantification, and a systematic review of clinical findings. Ageing Res Rev. 2017;36:50–63. 28315409 10.1016/j.arr.2017.03.002

[25] Nagaishi M, Yokoo H, Nakazato Y. Tau-positive glial cytoplasmic granules in multiple system atrophy. Neuropathol off J Jpn Soc Neuropathol. 2011;31:299–305. 10.1111/j.1440-1789.2010.01159.x 21062361

[26] Rong Z, Shen F, Wang Y, Sun L, Wu J, Zhang H, et al. Phosphorylated α-synuclein and phosphorylated tau-protein in sural nerves may contribute to differentiate Parkinson’s disease from multiple system atrophy and progressive supranuclear paralysis. Neurosci Lett. 2021;756:135964. 34022266 10.1016/j.neulet.2021.135964

[27] Vacchi E, Lazzarini E, Pinton S, Chiaro G, Disanto G, Marchi F, et al. Tau protein quantification in skin biopsies differentiates tauopathies from alpha-synucleinopathies. Brain J Neurol. 2022;145:2755–68. 10.1093/brain/awac161 35485527

[28] van Dyck CH, Swanson CJ, Aisen P, Bateman RJ, Chen C, Gee M, et al. Lecanemab in Early Alzheimer’s Disease. N Engl J Med. 2023;388:9–21. 36449413 10.1056/NEJMoa2212948

[29] Angioni D, Hansson O, Bateman RJ, Rabe C, Toloue M, Braunstein JB, et al. Can we use blood biomarkers as Entry Criteria and for Monitoring Drug Treatment effects in clinical trials? A report from the EU/US CTAD Task Force. J Prev Alzheimers Dis. 2023;10:418–25. 37357282 10.14283/jpad.2023.68

[30] Knopman DS, Amieva H, Petersen RC, Chételat G, Holtzman DM, Hyman BT, et al. Alzheimer disease. Nat Rev Dis Primer. 2021;7:33. 10.1038/s41572-021-00269-y PMC8574196 33986301

[31] Jack CR, Bennett DA, Blennow K, Carrillo MC, Dunn B, Haeberlein SB, et al. NIA-AA Research Framework: toward a biological definition of Alzheimer’s disease. Alzheimers Dement J Alzheimers Assoc. 2018;14:535–62. 10.1016/j.jalz.2018.02.018 PMC5958625 29653606

[32] NIA-AA Revised Clinical Guidelines for Alzheimer’sAAIC (//aaic.alz.org/nia-aa.asp.

[33] Boccalini C, Ribaldi F, Hristovska I, Arnone A, Peretti DE, Mu L et al. The impact of tau deposition and hypometabolism on cognitive impairment and longitudinal cognitive decline. Alzheimers Dement J Alzheimers Assoc. 2024;20:221–33. 10.1002/alz.13355 PMC10916991 37555516

[34] Orrú CD, Yuan J, Appleby BS, Li B, Li Y, Winner D, et al. Prion seeding activity and infectivity in skin samples from patients with sporadic Creutzfeldt-Jakob disease. Sci Transl Med. 2017;9:eaam7785. 29167394 10.1126/scitranslmed.aam7785 PMC5744860

[35] Wang Z, Manca M, Foutz A, Camacho MV, Raymond GJ, Race B, et al. Early preclinical detection of prions in the skin of prion-infected animals. Nat Commun. 2019;10:247. 30651538 10.1038/s41467-018-08130-9 PMC6335425

[36] Bargar C, Wang W, Gunzler SA, LeFevre A, Wang Z, Lerner AJ, et al. Streamlined alpha-synuclein RT-QuIC assay for various biospecimens in Parkinson’s disease and dementia with Lewy bodies. Acta Neuropathol Commun. 2021;9:62. 33827706 10.1186/s40478-021-01175-w PMC8028088

[37] Donadio V, Wang Z, Incensi A, Rizzo G, Fileccia E, Vacchiano V, et al. In vivo diagnosis of synucleinopathies: a comparative study of skin biopsy and RT-QuIC. Neurology. 2021;96:e2513-2524. 33837116 10.1212/WNL.0000000000011935 PMC8205473

[38] Kuzkina A, Bargar C, Schmitt D, Rößle J, Wang W, Schubert A-L, et al. Diagnostic value of skin RT-QuIC in Parkinson’s disease: a two-laboratory study. NPJ Park Dis. 2021;7:99. 10.1038/s41531-021-00242-2 PMC8593128 34782640

[39] Kuzkina A, Rößle J, Seger A, Panzer C, Kohl A, Maltese V, et al. Combining skin and olfactory α-synuclein seed amplification assays (SAA)-towards biomarker-driven phenotyping in synucleinopathies. NPJ Park Dis. 2023;9:79. 10.1038/s41531-023-00519-8 PMC10226020 37248217

[40] Chen D-D, Jiao L, Huang Y, Xiao K, Gao L-P, Chen C, et al. Application of α-Syn Real-Time Quaking-Induced Conversion for Brain and skin specimens of the Chinese patients with Parkinson’s Disease. Front Aging Neurosci. 2022;14:898516. 35847665 10.3389/fnagi.2022.898516 PMC9283982

[41] Mammana A, Baiardi S, Quadalti C, Rossi M, Donadio V, Capellari S, et al. RT-QuIC detection of pathological α-Synuclein in skin punches of patients with Lewy Body Disease. Mov Disord off J Mov Disord Soc. 2021;36:2173–7. 10.1002/mds.28651 PMC8518528 34002890

[42] Mammana A, Baiardi S, Rossi M, Franceschini A, Donadio V, Capellari S, et al. Detection of prions in skin punch biopsies of Creutzfeldt-Jakob disease patients. Ann Clin Transl Neurol. 2020;7:559–64. 32141717 10.1002/acn3.51000 PMC7187701

[43] Xiao K, Yang X, Zhou W, Chen C, Shi Q, Dong X. Validation and application of skin RT-QuIC to patients in China with probable CJD. Pathog Basel Switz. 2021;10:1642. 10.3390/pathogens10121642 PMC8707901 34959597

[44] Makrantonaki E, Brink TC, Zampeli V, Elewa RM, Mlody B, Hossini AM, et al. Identification of biomarkers of human skin ageing in both genders. Wnt signalling - a label of skin ageing? PLoS One. 2012;7:e50393. 23226273 10.1371/journal.pone.0050393 PMC3511529

[45] Miklossy J, Taddei K, Martins R, Escher G, Kraftsik R, Pillevuit O, et al. Alzheimer disease: curly fibers and tangles in organs other than brain. J Neuropathol Exp Neurol. 1999;58:803–14. 10446805 10.1097/00005072-199908000-00003

[46] Buée L, Bussière T, Buée-Scherrer V, Delacourte A, Hof PR. Tau protein isoforms, phosphorylation and role in neurodegenerative disorders. Brain Res Brain Res Rev. 2000;33:95–130. 10967355 10.1016/s0165-0173(00)00019-9

[47] Dugger BN, Hoffman BR, Scroggins A, Serrano GE, Adler CH, Shill HA, et al. Tau immunoreactivity in peripheral tissues of human aging and select tauopathies. Neurosci Lett. 2019;696:132–9. 30579993 10.1016/j.neulet.2018.12.031 PMC7357994

[48] Goedert M, Spillantini MG, Crowther RA. Cloning of a big tau microtubule-associated protein characteristic of the peripheral nervous system. Proc Natl Acad Sci U S A. 1992;89:1983–7. 1542696 10.1073/pnas.89.5.1983 PMC48578

[49] Hamsafar Y, Chen Q, Borowsky AD, Beach TG, Serrano GE, Sue LI, et al. Biochemical analyses of tau and other neuronal markers in the submandibular gland and frontal cortex across stages of Alzheimer disease. Neurosci Lett. 2023;810: 137330. 37330193 10.1016/j.neulet.2023.137330 PMC11006283

[50] Akerman SC, Hossain S, Shobo A, Zhong Y, Jourdain R, Hancock MA, et al. Neurodegenerative disease-related proteins within the epidermal layer of the human skin. J Alzheimers Dis JAD. 2019;69:463–78. 31006686 10.3233/JAD-181191

[51] Dugger BN, Whiteside CM, Maarouf CL, Walker DG, Beach TG, Sue LI, et al. The Presence of Select Tau species in Human Peripheral tissues and their relation to Alzheimer’s Disease. J Alzheimers Dis JAD. 2016;51:345–56. 26890756 10.3233/JAD-150859 PMC6074044

[52] Rodriguez-Leyva I, Chi-Ahumada E, Calderon A, Laura A, Verónica M-M. E S, Presence of phosphorylated tau protein in the skin of alzheimer´s disease patients. J Mol Biomark Diagn. 2015;S6:005.

[53] Iranzo A, Mammana A, Muñoz-Lopetegi A, Dellavalle S, Mayà G, Rossi M, et al. Misfolded α-Synuclein Assessment in the skin and CSF by RT-QuIC in isolated REM sleep behavior disorder. Neurology. 2023;100:e1944-54. 36931726 10.1212/WNL.0000000000207147 PMC10159765

[54] Manne S, Kondru N, Jin H, Anantharam V, Huang X, Kanthasamy A, et al. α-Synuclein real-time quaking-induced conversion in the submandibular glands of Parkinson’s disease patients. Mov Disord off J Mov Disord Soc. 2020;35:268–78. 10.1002/mds.27907 PMC7102508 31758740

[55] Zhang W, Orrú CD, Foutz A, Ding M, Yuan J, Shah SZA, et al. Large-scale validation of skin prion seeding activity as a biomarker for diagnosis of prion diseases. Acta Neuropathol. 2024;147:17. 38231266 10.1007/s00401-023-02661-2 PMC11812622

[56] Altamirano-Bustamante MM, Altamirano-Bustamante NF, Larralde-Laborde M, Lara-Martínez R, Leyva-García E, Garrido-Magaña E, et al. Unpacking the aggregation-oligomerization-fibrillization process of naturally-occurring hIAPP amyloid oligomers isolated directly from sera of children with obesity or diabetes mellitus. Sci Rep. 2019;9:18465. 31804529 10.1038/s41598-019-54570-8 PMC6895187

[57] Bittar A, Bhatt N, Hasan TF, Montalbano M, Puangmalai N, McAllen S, et al. Neurotoxic tau oligomers after single versus repetitive mild traumatic brain injury. Brain Commun. 2019;1:fcz004. 31608324 10.1093/braincomms/fcz004 PMC6777515

[58] Metrick MA, Ferreira N do, Saijo C, Kraus E, Newell A, Zanusso K. A single ultrasensitive assay for detection and discrimination of tau aggregates of Alzheimer and pick diseases. Acta Neuropathol Commun. 2020;8:22. 32087764 10.1186/s40478-020-0887-z PMC7036215

[59] Zou W-Q, Zheng J, Gray DM, Gambetti P, Chen SG. Antibody to DNA detects scrapie but not normal prion protein. Proc Natl Acad Sci U S A. 2004;101:1380–5. 14734804 10.1073/pnas.0307825100 PMC337061

[60] Chang E, Kuret J. Detection and quantification of tau aggregation using a membrane filter assay. Anal Biochem. 2008;373:330–6. 17949677 10.1016/j.ab.2007.09.015 PMC2359897

[61] Zou WQ, Yang DS, Fraser PE, Cashman NR, Chakrabartty A. All or none fibrillogenesis of a prion peptide. Eur J Biochem. 2001;268:4885–91. 11559357 10.1046/j.1432-1327.2001.02415.x

