# Supplementary material for: Seeding activity of skin misfolded tau as a biomarker for tauopathies
Source: Mol Neurodegener. 2024 Nov 29;19:92. doi: 10.1186/s13024-024-00781-1 (PMC11606191; doi:10.1186/s13024-024-00781-1)
Supplement: Supplementary file 1 — Supplementary Material 1. [file 13024_2024_781_MOESM1_ESM.docx]

Seeding Activity of Skin Misfolded Tau as a Biomarker for Tauopathies

**Authors:**

Zerui Wang^1*^, Ling Wu^2^, Maria Gerasimenko^1^, Tricia Gilliland^1^, Zahid Syed Ali Shah^1^, Evalynn Lomax^1^, Yirong Yang^3^, Steven A. Gunzler^4,5^, Vincenzo Donadio^6^, Rocco Liguori^6^, Bin Xu^2*^, Wen-Quan Zou ^1,3,5*^

**Affiliations:**

^1^Department of Pathology, Case Western Reserve University School of Medicine; Cleveland, Ohio, USA.

^2^Biomanufacturing Research Institute and Technology Enterprise, Durham, North Carolina Central University; Durham, North Carolina, USA.

^3^Institute of Neurology, Department of Neurology, Jiangxi Academy of Clinical Medical Sciences, Rare Disease Center, Key Laboratory of Rare Neurological Diseases of Jiangxi Province Health Commission, The First Affiliated Hospital, Jiangxi Medical College, Nanchang University, Nanchang, Jiangxi Province, China.

^4^Neurological Institute, University Hospitals Cleveland Medical Center; Cleveland, Ohio, USA.

^5^Department of Neurology, University Hospitals Cleveland Medical Center and Case Western Reserve University School of Medicine; Cleveland, Ohio, USA.

^6^IRCCS Institute of Neurological Sciences of Bologna, UOC Clinica Neurologica; Bologna, Italy.

*Wen-Quan Zou, Email: [wenquanzou@nuc.edu.cn](mailto:wenquanzou@nuc.edu.cn); or *Bin Xu, Email: [bxu@nccu.edu](mailto:bxu@nccu.edu); or *Zerui Wang, Email: zxw488@case.edu

**This file includes:**

Supplementary Text

Figs. S1 to S6

**Supplementary figures and figure legends**


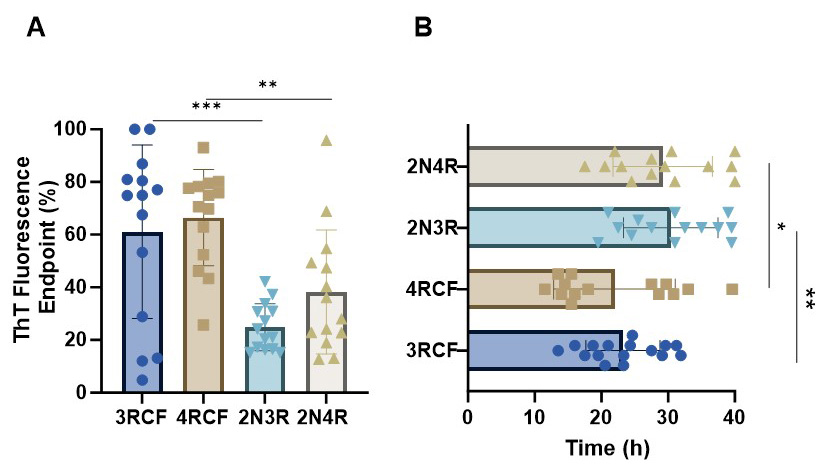
Figure S1

**Fig. S1. Assessment of the full-length and truncated tau as the substrate of skin tau RT-QuIC assay.** (**A**) Comparing the endpoint tau-SAA fluorescence intensity between the truncated (4RCF/3RCF tau fragments) and full-length (2N3R/2N4R) tau substrate with skin tissues from AD cadavers diagnosed neuropathologically (n = 14) (**B**) Comparing the tau-SAA lag phase results between truncated (4RCF/3RCF) and full-length (2N3R/2N4R) tau substrates with skin tissues as mentioned in **A**. The lag phase was determined based on the time period from the beginning of the RT-QuIC assay to starting to an increase in the kinetic ThT fluorescence curve. *: *p* < 0.05; **: *p* < 0.01; ***: *p* < 0.001.


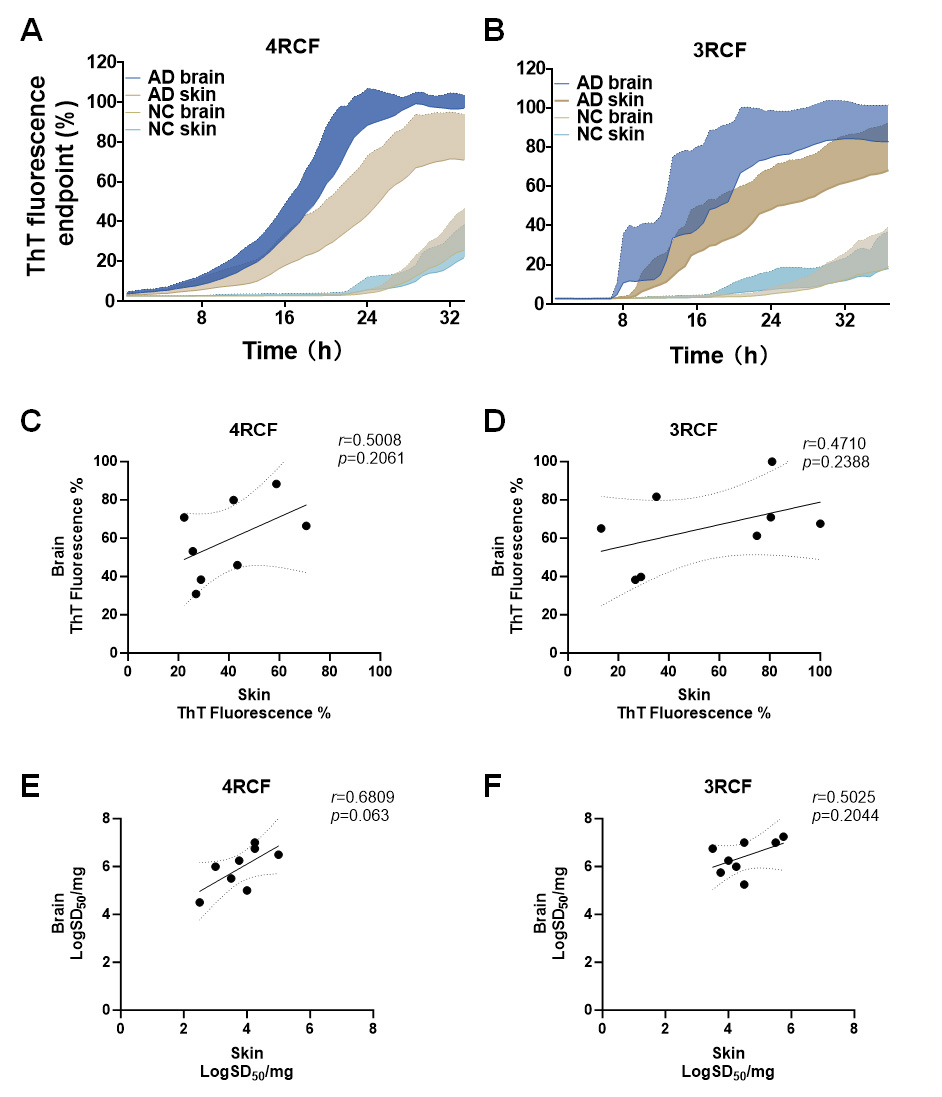
Figure S2

**Fig. S2. Comparison of tau-SA between the brain and skin tissues from AD and non-AD cases.** Kinetic curves representing average tau-SA with 4RCF (**A**) or with 3RCF (**B**) as the substrate and standard deviation over time in the brain (n = 12) and skin samples (n = 20) of AD cases and the brain and skin samples (20 each) of normal controls (NC). **C**-**F**: The correlation analysis of the ThT fluorescence intensity between matching brain and skin (n=8) with 4RCF (**C**) and 3RCF (**D**), and correlation analysis of the SD50 between matching brain and skin (n=8) with 4RCF (**E**) and 3RCF (**F**)

Figure S3

**Fig. S3. Comparative western blotting of AD brain and skin samples.** Representative western blotting of AD brain (n = 6) and skin samples (n =7) probed with anti-phosphorylated tau antibody pT231 (**A**) and pS396 (**B**) for the comparison of the brain and skin tau. Representative western blotting of skin samples from AD cadavers (n = 7) probed with anti-tau antibody RD3 (**C**) and RD4 (**D**). Based on densitometric analysis of the panels **C** and **D**, the ratio of 3R/4R of AD skin samples used in our study was measured and it was approximately 1.3:1. Quantitative analysis of the pTau value in the AD brain and tauopathies’ skin as shown in **E** (pT231) and **F** (pS396)

Figure S4


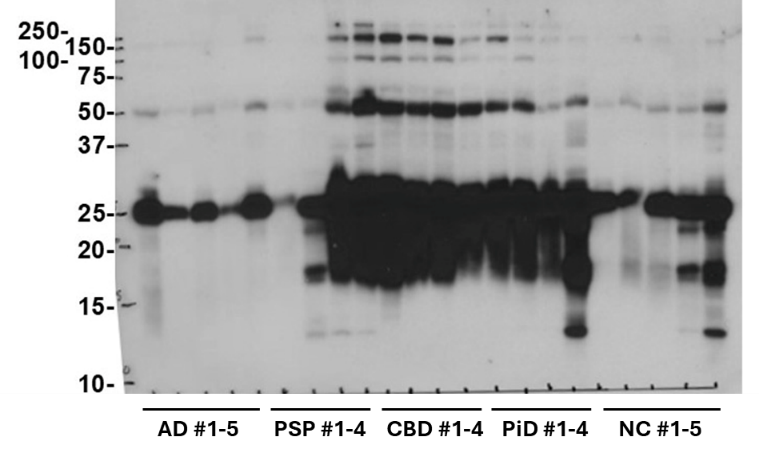


**Fig. S4. Western blot of tau in the skin of cadavers with various tauopathies.** Representative western blotting of skin homogenates from AD (n = 5), PSP (n = 4), CBD (n = 4), PiD (n = 4) and NC (n = 5), probed by the anti-tau antibody HT7.

Figure S5

**Fig. S5. RT-QuIC assay of AD skin samples with or without immunodepletion with anti-tau antibody.** Skin homogenates from AD cadavers subjected to with immunoprecipitation (IP) with anti-tau antibody Tau5 [immunodepletion (ID) (n = 10) or not subject to ID (n = 10) before RT-QuIC assay. The tau-SA was significantly decreased in samples treated with ID than samples without ID (mean ± SD, p < 0.001).

Figure S6

**Fig. S6. Effect of age, gender and PMI on skin tau-SA.** (**A**) Scatter plot shows gender-adjusted ThT fluorescence in AD autopsy skin samples using 4RCF as RT-QuIC substrate with no significant difference between groups (*p* > 0.05). (**B**) Scatter plot reveals that gender-adjusted ThT fluorescence in PSP autopsy skin samples using 4RCF method with no significant difference between groups (*p* > 0.05). (**C**) Gender-adjusted ThT fluorescence in AD autopsy skin samples using 3RCF as substrate indicates no significant difference between groups (*p* > 0.05). (**D**) Gender-adjusted ThT fluorescence in PSP autopsy skin samples using 3RCF as substrate demonstrating no significant difference between groups (*p* > 0.05). (**E**) Age correlation with ThT fluorescence across all tauopathies using 4RCF as substrate: weak positive correlation (r = 0.07247), not statistically significant (*p* > 0.05). (**F**) PMI correlation with ThT fluorescence across all tauopathies using 4RCF as substrate: weak negative correlation (r = -0.02187), not statistically significant (*p* > 0.05). (**G**) Age correlation with ThT fluorescence across all tauopathies using 3RCF as the substrate: slightly positive (r = 0.1042), not statistically significant (*p* > 0.05). (**H**) PMI correlation with ThT fluorescence across all tauopathies using 3RCF as the substrate: slightly negative (r = -0.1225), not statistically significant (*p* > 0.05).
